# Luminescent Chiral Molecular Glasses by Melt‐Quenching Enantiopure BINAP

**DOI:** 10.1002/advs.202518879

**Published:** 2025-11-23

**Authors:** Nuttaporn Krittametaporn, Philipp Ralle, Dorothea Dierks, Christian Nelle, Suresh K. Vasa, Pascal Kolodzeiski, Rasmus Linser, Andreas Steffen, Sebastian Henke

**Affiliations:** ^1^ Anorganische Chemie, Fakultät für Chemie und Chemische Biologie Technische Universität Dortmund Otto‐Hahn‐Straße 6 44227 Dortmund Germany; ^2^ Physikalische Chemie, Fakultät für Chemie und Chemische Biologie Technische Universität Dortmund Otto‐Hahn‐Straße 4a 44227 Dortmund Germany

**Keywords:** amorphous materials, circularly polarized luminescence, melt‐quenching, molecular glasses, optical materials

## Abstract

Chiral organic glasses combine unique optical properties with the processing advantages of amorphous solids. Here, melt‐quenching as a strategy for preparing optically active glasses from enantiopure BINAP (2,2′‐bis(diphenylphosphino)‐1,1′‐binaphthyl), a pivotal ligand in asymmetric catalysis and for luminescent metal complexes is demonstrated. Thermal characterization reveals that only *R*‐BINAP and *S*‐BINAP, not *rac*‐BINAP, form molecular glasses with glass transition temperatures near 100 °C. Pair distribution function analysis and circular dichroism confirm the retention of local structure and homochirality despite the loss of long‐range order. Remarkably, the glassy state has a beneficial influence on the molecular optoelectronic properties relative to the crystalline state, resulting in an increase of the radiative rate constant by ≈30%, attributed to more favourable Franck‐Condon factors. In addition, a highly unusual simultaneous enhancement of circularly polarized luminescence (CPL) by nearly an order of magnitude is observed, achieving dissymmetry factors |*g*_lum_| approaching 10^−2^ that are competitive with the top‐performing purely organic molecular chiral emitters reported to date. These findings establish melt‐quenched chiral molecular glasses as promising platforms for advanced optoelectronic and photonic materials, combining exceptional chiroptical properties, strong luminescence, and processability without the constraints of crystallinity.

## Graphical abstract

Melt‐quenching of enantiopure BINAP yields transparent chiral molecular glasses that retain axial chirality and display blue‐shifted luminescence together with strongly enhanced circularly polarized emission with dissymmetry factors |g_lum_| of ≈10^−2^. These materials unite optical clarity, strong emission, and high chiroptical activity, highlighting vitrification as a powerful strategy for creating solid‐state chiral photonic materials.

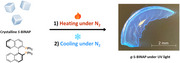

## 1 Introduction

Chiral organic materials have become central to modern optics and photonics, enabling the manipulation of circularly polarized light and providing new avenues for enantioselective photochemical processes.^[^ ^1^, ^2^ ^]^ Their growing importance is particularly evident in emerging fields such as quantum optics, metamaterials, and chiral sensing.^[^ ^3^, ^4^ ^]^ Among the various solid‐state forms, glasses offer unique advantages: isotropic optical properties, the absence of grain boundaries, and the potential for processing into complex geometries.^[^ ^5^, ^6^, ^7^, ^8^ ^]^ While the glass formation of achiral organic molecules has been widely studied,^[^ ^9^, ^10^, ^11^, ^12^, ^13^ ^]^ the interplay between molecular chirality and glass‐forming ability remains largely unexplored,^[^ ^13^, ^14^, ^15^, ^16^ ^]^ especially for technologically relevant systems.

Organic molecular glasses, metal‐organic glasses, and hybrid organic‐inorganic metal halide glasses exhibit thermal, mechanical, and optical properties distinct from their crystalline analogues.^[^ ^13^, ^14^, ^17^, ^18^, ^19^, ^20^ ^]^ The glass transition in small organic molecules is governed by molecular flexibility, intermolecular interactions, and packing efficiency – all factors that are strongly modulated by chirality.^[^ ^19^, ^21^, ^22^, ^23^ ^]^ Notably, chiral glasses can retain optical activity while circumventing issues of crystalline polymorphism and light‐scattering grain boundaries, presenting clear benefits for optoelectronic applications. Circularly polarized luminescence (CPL) is particularly valuable for photonic technologies,^[^ ^24^, ^25^ ^]^ yet reliable CPL measurements in crystals are often hampered by linear dichroism artefacts and grain boundary scattering, making isotropic chiral glasses attractive alternatives.^[^ ^26^ ^]^

2,2′‐Bis(diphenylphosphino)‐1,1′‐binaphthyl (BINAP) stands as a paradigmatic chiral molecule, renowned for its impact on asymmetric catalysis^[^ ^27^, ^28^, ^29^ ^]^ and luminescent transition metal complexes.^[^ ^30^, ^31^ ^]^ Its rigid binaphthyl backbone confers exceptional configurational stability (racemization barrier Δ*G* ^‡^ > 180 kJ mol^−1^),^[^ ^32^ ^]^ ensuring the retention of homochirality even at elevated temperatures. Crystalline BINAP exhibits distinct photophysical properties, including blue‐green fluorescence and room‐temperature phosphorescence, with racemic (*rac*‐)BINAP in particular displaying enhanced red phosphorescence due to its rigid, heterochiral crystal packing that suppresses non‐radiative decay pathways.^[^ ^1^, ^33^ ^]^

Despite BINAP's prominence and promise for chiral optical applications, its thermal behavior and glass‐forming ability have not been systematically investigated. Previously, powder forms of amorphous BINAP have been prepared through rapid solvent evaporation methods,^[^ ^33^ ^]^ but no studies have systematically investigated melt‐quenched glasses of BINAP, leaving an important gap in the understanding of their thermophysical behavior, structural characteristics and photophysical properties. This gap is significant given BINAP's rigid aromatic framework and strong π–π interactions, which may impose unusual constraints on crystallization and glass formation. Furthermore, comparative studies of racemic and enantiopure forms offer an opportunity to gain fundamental insights into the influence of chirality on glass forming behavior.

In this work, we present the first comprehensive study of melt‐quenched glass formation in BINAP, systematically comparing enantiopure *R*‐ and *S*‐BINAP with *rac*‐BINAP. Glass formation is observed only for the enantiopure forms as demonstrated by thermal analysis and X‐ray total scattering with pair distribution function (PDF) analysis, while complementary solid‐state NMR, IR spectroscopy, and optical methods (circular dichroism and CPL spectroscopy) provide insights into local structure and chiroptical properties. The resulting chiral BINAP glasses retain molecular chirality, exhibit distinctly blue‐shifted luminescence relative to the crystalline state, and display substantially enhanced dissymmetry in their emitted light. These findings establish BINAP glasses as processable, optically active materials with advantageous chiroptical properties, offering new opportunities for future solid‐state photonic technologies.

## 2 Results and Discussion

### 2.1 Glass Formation and Thermal Characterization

Microcrystalline samples of *R*‐BINAP, *S*‐BINAP and *rac*‐BINAP were obtained from commercial sources and confirmed to be of high purity by solution‐phase ^1^H and ^31^P NMR spectroscopy (Figures S1 to S3, Supporting Information). Profile fitting of powder X‐ray diffraction (PXRD) patterns (Pawley method) confirmed that all three derivatives are phase‐pure and consistent with previously reported crystallographic data (Figures S4 to S6, Supporting Information).^[^ ^33^ ^]^ Enantiopure *R*‐ and *S*‐BINAP crystallize in the monoclinic space group *P*2_1_ with determined crystallographic densities of 1.23 and 1.22 g cm^−3^, respectively (Table S1, Supporting Information). In contrast, *rac*‐BINAP adopts a racemic crystal structure in space group *C*2/*c*, with a higher density of 1.26 g cm^−3^, reflecting the more efficient molecular packing characteristic of the racemic crystal structure, in agreement with earlier reports.^[^ ^33^ ^]^ Thermogravimetric and differential thermal analysis (TG/DTA) conducted in open crucibles under constant N_2_ flow with a heating rate of 10 °C min^−1^ reveal that all BINAP forms begin to volatize at ≈350 °C. Complete mass loss was observed for all samples by 500 °C, confirming that no thermal decomposition occurs during sublimation (Figure S7, Supporting Information).

**Figure 1:**
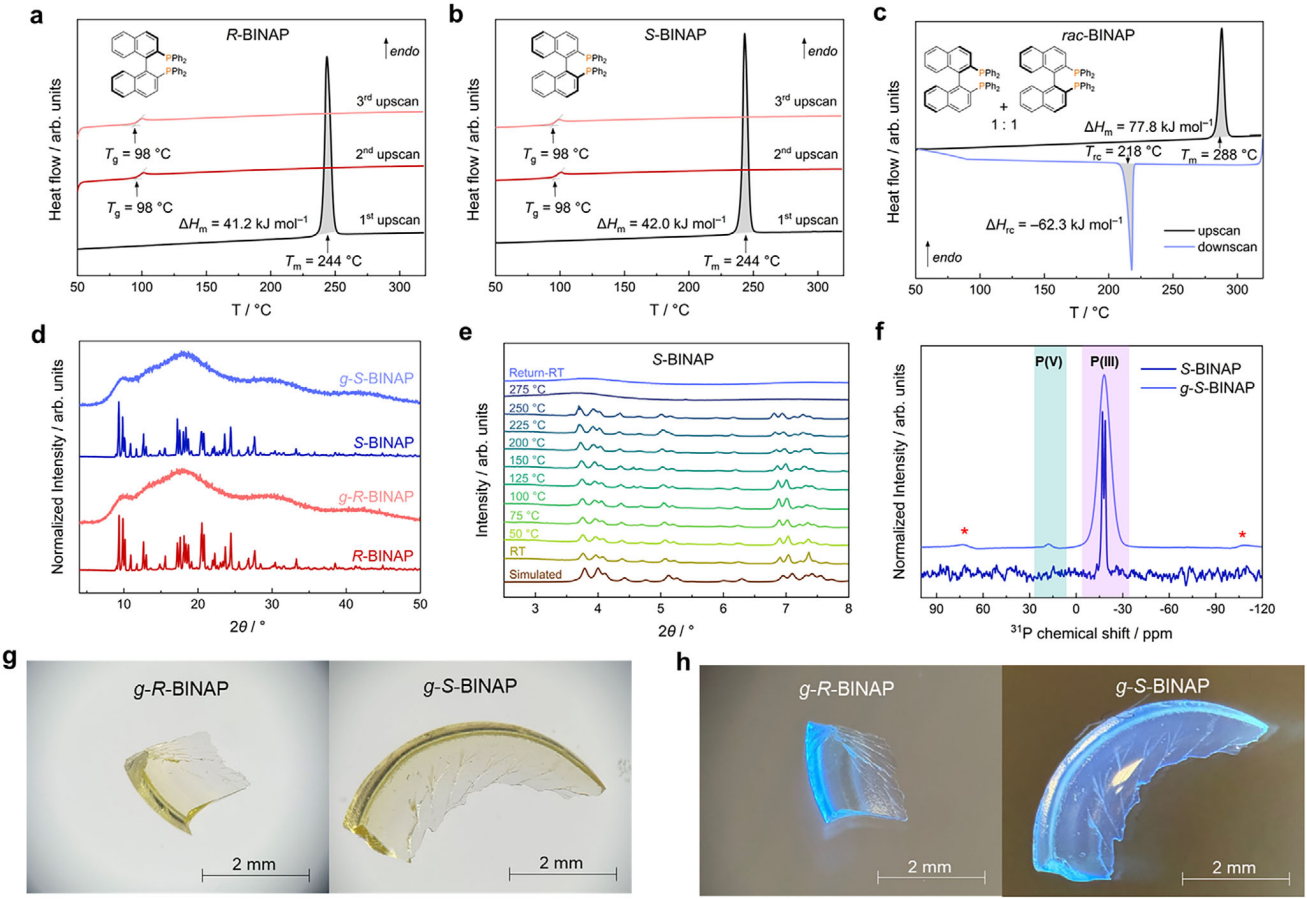
DSC data of a) *R*‐BINAP, b) *S*‐BINAP, and c) *rac*‐BINAP recorded under N_2_ atmosphere with heating/cooling rates of ±10 °C min^−1^. d) PXRD patterns of the crystalline and glassy states of *R*‐BINAP and *S*‐BINAP. The patterns are vertically offset for clarity. e) VT‐PXRD patterns of *S*‐BINAP from room temperature (RT) to 275 °C, including another pattern after returning to ambient conditions (λ = 0.61992 Å). The patterns are vertically offset for clarity. The simulated pattern was calculated based on crystallographic data from the literature (CCDC 1968819).^[^ ^33^ ^]^ f) Comparison of ^31^P MAS NMR spectra for *S*‐BINAP in its crystalline and glass forms. The highlighted regions indicate signals corresponding to P(V) and P(III) species. Signals marked with red * represent spinning sidebands. Micrographs of *g*‐*R*‐BINAP and *g*‐*S*‐BINAP under g) visible light and h) UV light.

Cyclic DSC measurements (two upscans, one downscan) were conducted on all BINAP derivatives using hermetically sealed aluminum crucibles prepared under an inert Ar atmosphere. The samples were heated from room temperature to 320 at 10 °C min^−1^ under N_2_ flow to assess melting and glass formation. The enantiomers *R*‐ and *S*‐BINAP exhibit sharp endothermic melting peaks at ≈244 °C (*T*_m_ = melting temperature), with closely matching melting enthalpies (Δ*H*_m_) of 41.2 kJ mol^−1^ (*R*‐BINAP) and 42.0 kJ mol^−1^ (*S*‐BINAP) (**Figure** **1**a,b). In comparison, *rac*‐BINAP displays a markedly higher *T*_m_ of 288 °C and a Δ*H*_m_ of 77.8 kJ mol^−1^ (Figure 1c). The assignment of these thermal events to melting, rather than to solid‐state phase transitions, is supported by variable temperature (VT‐)PXRD, which reveals a complete loss of Bragg reflections for *R*‐ and *S*‐BINAP above 250 °C (Figures 1e; S9, Supporting Information). The pronounced difference in both melting temperature and enthalpy between racemic and enantiopure BINAP is consistent with the higher packing efficiency of the racemic phase and reflects the remarkably higher thermodynamic stability of the racemic crystal form compared to the enantiopure ones.

Upon cooling the melt at a rate of –10 °C min^−1^, *rac*‐BINAP exhibits pronounced recrystallization, with an exothermic peak centered at 218 °C and a recrystallization enthalpy (Δ*H*_rc_) of –62.3 kJ mol^−1^ (Figures 1c; S10, Supporting Information). No indications of glass formation are observed in the DSC trace, and a second heating scan reveals remelting at the same *T*_m_ as in the initial upscan. This behavior indicates the strong tendency of *rac*‐BINAP to return to the crystalline state upon cooling. In contrast, the enantiopure forms, *R*‐ and *S*‐BINAP, display no exothermic crystallization signals upon cooling from 320 to 30 °C. This is further corroborated by VT‐PXRD, which reveals no Bragg reflections after cooling from 275 °C to room temperature. In subsequent heating scans, both chiral BINAP derivatives show clear glass transition signals at *T*_g_ = 98 °C, confirming successful vitrification under these conditions (Figures 1a,b; S11, Supporting Information). The resulting chiral glasses, denoted *g*‐*R*‐BINAP and *g*‐*S*‐BINAP, form monolithic, transparent bulk samples following fusion of the microcrystalline particles during melting. The glasses can be readily isolated as millimeter‐sized, pale‐yellow transparent shards from the DSC pans (Figure 1g). PXRD analysis further confirms the absence of crystalline order, as indicated by the sole presence of broad diffuse scattering features in the PXRD patterns of *g*‐*R*‐BINAP and *g*‐*S*‐BINAP (Figure 1d).

Remarkably, heating of the glasses above their *T*_g_ to 320 °C does not induce crystallization of the supercooled liquid, and a subsequent DSC heating scan again reveals a *T*_g_ signal at 98 °C, demonstrating the high resistance of the enantiopure BINAP glasses to recrystallization even at temperatures approaching *T*_m_. This contrasts sharply with enantiopure BINAP glasses prepared by rapid solvent evaporation, which were reported to readily recrystallize upon heating to 150 °C.^[^ ^33^ ^]^ To further assess the kinetic stability of these glasses, additional cyclic DSC experiments at varying cooling and heating rates were conducted to determine the dimensionless calorimetric fragility index (*m*), based on the rate‐dependent shift in the fictive temperature (*T*_f_, see Supplementary Information for details). The fragility index quantifies how rapidly the viscosity changes with temperature near *T*_g_.^[^ ^17^, ^34^ ^]^ Strong glass formers, which retain high viscosity in the supercooled liquid, exhibit low fragility indices, whereas poor glass formers, whose viscosity decreases rapidly above *T*_g_, display high fragility indices. For reference, a typical strong glass former such as SiO_2_ has a fragility index of ≈20.^[^ ^35^ ^]^ For *g*‐*R*‐BINAP and *g*‐*S*‐BINAP, *m* values of 52.5 ± 6.1 and 50.6 ± 5.3, respectively, were obtained (Figures S12 and S13, Supporting Information), indicating intermediate fragility and a correspondingly moderate temperature dependence of viscosity in the supercooled liquid state, correlating with the pronounced stability against crystallization observed in our experiments.

Solid‐state ^13^C and ^31^P magic angle spinning (MAS) NMR spectra were recorded for microcrystalline *S*‐BINAP as well as its corresponding glass (Figures 1f; S14 and S15, Supporting Information). The crystalline material features two sharp ^31^P resonances at –16.91 and –18.62 ppm, consistent with the presence of two crystallographically distinct phosphorus atoms in the asymmetric unit, as expected from the known crystal structure. In contrast, the ^31^P MAS NMR spectrum of *g*‐*S*‐BINAP displays a single broad resonance centered at –17.70 ppm, indicative of pronounced structural disorder and a broad distribution of phosphorous environments in the glassy state. Additionally, a weak broad signal located at 18.23 ppm, characteristic of P(V) species, is observed. This finding suggests minor partial oxidation of *S*‐BINAP (presumably to BINAP monoxide and BINAP dioxide) during melting and glass formation, despite all thermal treatment being conducted under inert atmosphere. Quantitative integration of the P(III) and P(V) signals indicates a total P(V) content of ≈0.9%, confirming that oxidized species remain a minor impurity in the glass. Partial oxidation to P(V) is further corroborated by solution‐phase ^1^H and ^31^P NMR spectra of *g‐R*‐BINAP and *g‐S*‐BINAP dissolved in THF‐*d*_8_, which also reveal minor resonances attributed to P(V) species (Figures S16 and S17, Supporting Information). Notably, the fraction of oxidized species detected by solution‐phase ^31^P NMR is higher than that observed in the solid‐state spectra, suggesting that the glassy BINAP materials are more susceptible to oxidation upon dissolution compared to their crystalline counterparts.

### 2.2 Local Structure Analysis

**Figure 2:**
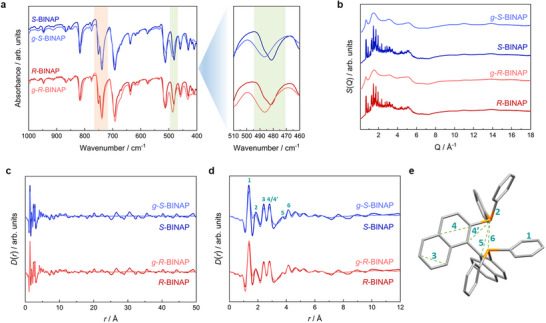
a) Comparison of FTIR spectra for crystalline *R*‐BINAP and *S*‐BINAP and their corresponding glasses in the range of 400 to 1000 cm^−1^. The region of the aromatic C–H out‐of‐plane bending modes is highlighted in light red. The graph on the right emphasizes the shift of the phenyl out‐of‐plane ring vibration, highlighted in light green. b) Total scattering functions *S*(*Q*) collected for crystalline *R*‐BINAP and *S*‐BINAP, and their glasses. c) PDFs in the form *D*(*r*) over the *r*‐range from 0 to 50 Å. d) Zoom into the short‐ to medium‐range distances of the PDFs. e) Illustration of the key short‐range atomic distances in an *S*‐BINAP molecule, based on the reported crystal structure (CCDC: 1968819).^[^ ^33^ ^]^ C and P atoms are shown in gray and orange. H atoms are omitted.

To probe structural changes accompanying the crystalline‐to‐glass transition in *R*‐BINAP and *S*‐BINAP, Fourier‐transform infrared (FTIR) spectra were recorded for both crystalline and glassy forms. The FTIR spectra of the crystalline enantiopure BINAP samples are nearly identical and align with the previous literature (**Figures** **2**a; S18, Supporting Information),^[^ ^36^ ^]^ reflecting the close similarity in their molecular structures and packing arrangements. Upon vitrification, the FTIR spectra of *g*‐*R*‐BINAP and *g*‐*S*‐BINAP exhibit pronounced band broadening, characteristic of increased structural disorder. This effect is especially evident in the region between 720 and 760 cm^−1^, where two distinct bands present in the crystalline phases merge into a single broad feature in the glasses. Density functional theory (DFT) calculations assign these bands to aromatic C–H out‐of‐plane bending modes of the phenyl and naphthyl groups. Their broadening and merging confirm the presence of a wide distribution of local environments and disordered molecular packing in the glassy state. In the lower‐frequency region, the phenyl out‐of‐plane ring deformation mode shifts from 481 to 487 cm^−1^ upon vitrification (Figure 2a). The 6 cm^−1^ blueshift indicates a slightly stiffer local potential and/or reduced lattice coupling for this specific vibration in the glasses compared to the crystalline phases, pointing to weaker or less coherent π···π and C–H···π interactions in the glassy state in line with the loss of long‐range order.

Further insights into the local and intermediate‐range structure were obtained from X‐ray total scattering measurements performed on both crystalline and glassy forms of *R*‐BINAP and *S*‐BINAP. The total scattering functions *S*(*Q*) of the melt‐quenched glasses lack any traces of Bragg reflections, confirming the complete absence of crystalline domains. Peak assignments in the PDFs, derived via Fourier transformation of *S*(*Q*), were guided by the known crystal structure of *S*‐BINAP. For crystalline BINAP, the PDFs (*D*(*r*)) exhibit strong atom pair correlations up to ≈6 Å, followed by weaker correlations up to at least 50 Å, reflecting the periodic molecular packing. In contrast, these longer‐range correlations vanish in the PDFs of the glassy phases, providing direct evidence for the absence of long‐range structural order (Figure 2c). A detailed comparison at short distances (*r* < 12 Å) reveals that the PDFs of both phases are very similar (Figure 2d). However, the glassy phases exhibit broader features and slight peak shifts in the 1–6 Å region, indicating subtle variations in local molecular environments and conformations. By contrast, the prominent correlation at 4 Å, corresponding to the intramolecular P···P distance within a BINAP molecule (see distance 6 in Figure 2e), remains unchanged after vitrification. Together, these findings show that glass formation disrupts long‐range packing while largely preserving the intramolecular conformation of BINAP, with structural disorder primarily confined to intermolecular arrangements.

### 2.3 Optical and Photophysical Properties

**Figure 3:**
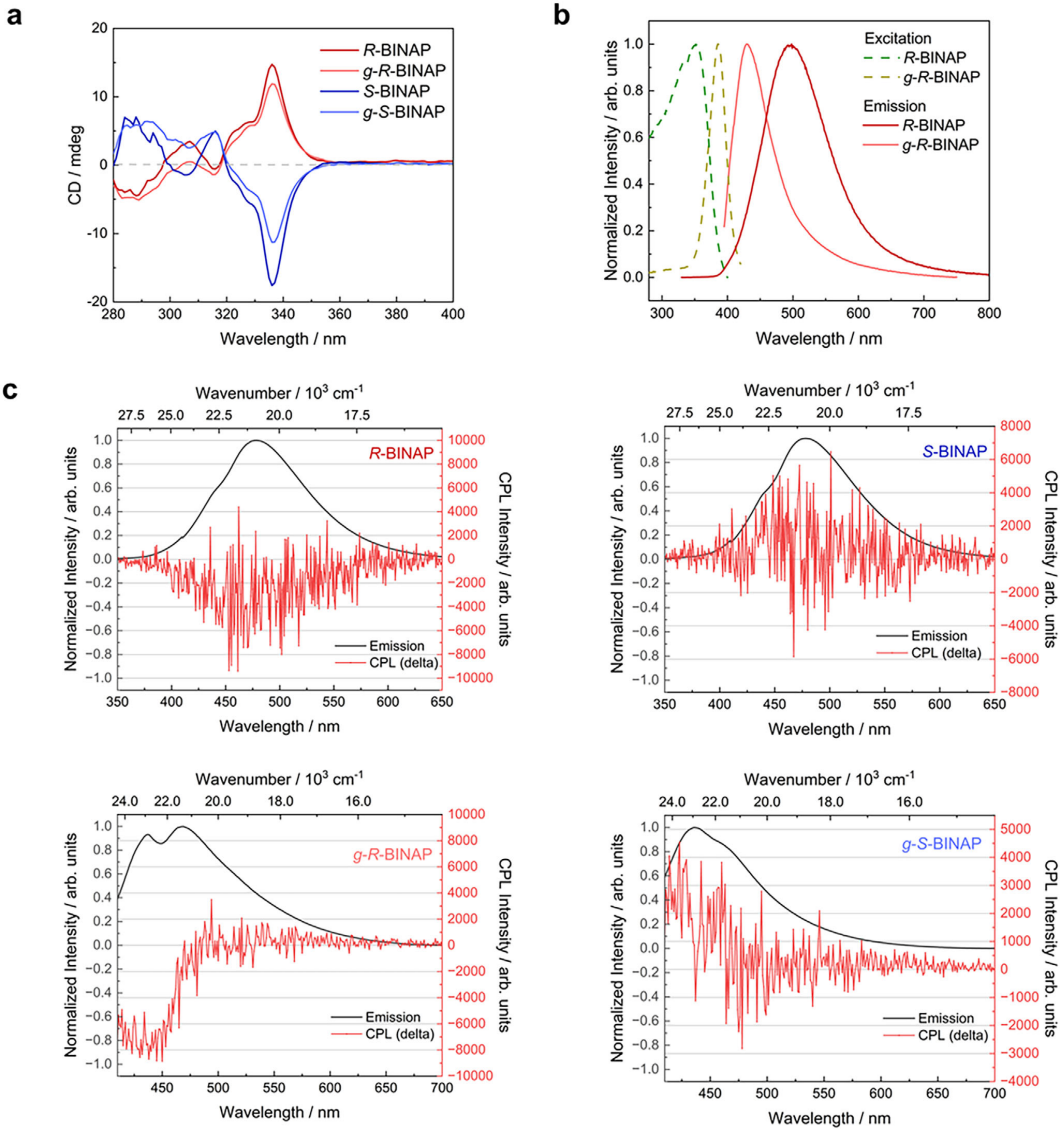
a) CD spectra of *R*‐ and *S*‐BINAP in their crystalline and glassy forms, measured after dissolution in CHCl_3_ (concentration: 2 × 10^−4^ M). b) Solid‐state excitation and emission spectra of *R*‐BINAP and *g*‐*R*‐BINAP. c) Solid‐state CPL spectra (unsmoothed) recorded at room temperature for *R*‐BINAP, *S*‐BINAP, *g*‐*R*‐BINAP and *g*‐*S*‐BINAP, respectively.

To confirm the retention of homochirality and enantiomeric purity after glass formation, CD spectroscopy was performed on solutions of crystalline and glassy *R*‐BINAP and *S*‐BINAP in CHCl_3_. In solution, both crystalline‐derived and glass‐derived samples exhibit strong, nearly perfect mirror‐image Cotton effects across the 280–400 nm range, with a pronounced feature centered at 338 nm corresponding to π–π* transitions within the BINAP aromatic backbone (**Figure** **3**a).^[^ ^33^, ^37^ ^]^ The spectra of the solutions prepared from the crystalline materials are consistent with established literature data,^[^ ^33^, ^37^ ^]^ and the solutions of the corresponding glassy materials show very similar overall profiles. This confirms that the melt‐quenched materials remain optically active. However, a modest reduction in ellipticity is observed for the glass‐derived samples compared to those derived from the crystalline counterparts. This decrease may reflect a slight loss of chirality, either through partial racemization during high‐temperature melting or the formation of small amounts of achiral or racemic P(V) species, as indicated by NMR data. Nevertheless, the characteristic solution CD responses of the melt‐quenched glasses highlight that optical activity is largely preserved in *g*‐*R*‐BINAP and *g*‐*S*‐BINAP.

Given the structural similarity of BINAP enantiomers, solid‐state excitation and emission spectra were recorded for *R*‐BINAP in both crystalline and glassy forms (Figure 3b). The lowest energy excitation of *g*‐*R*‐BINAP is red‐shifted to ≈380 nm and notably sharper than that of the crystalline material, which exhibits a broader band centered at 350 nm, which is in line with the reported samples of crystalline BINAP.^[^ ^33^ ^]^ As the glass is a kinetically trapped, higher‐enthalpy state rather than a thermodynamically favored phase, the molecular ground state of *g*‐*R*‐BINAP experiences different intermolecular interactions and is thus destabilized relative to the crystalline phase. This destabilization, in combination with subtle local dielectric effects in the disordered matrix, decreases the excitation energy gap S_0_→S_1_ between the ground state and the first electronically excited singlet state and leads to the observed bathochromic shift.^[^ ^30^, ^38^ ^]^

Both materials exhibit intense visible emission, but with marked differences in spectral position and shape. While crystalline *R*‐BINAP shows a broad emission centered at 498 nm with FWHM*_R_*_‐BINAP_ ≈ 4720 cm^−1^, *g*‐*R*‐BINAP emits surprisingly hypsochromically shifted with λ_max_ = 430 nm and a narrower band (FWHM*_g_*_‐_ *_R_*_‐BINAP_ ≈ 4130 cm^−1^). In addition, the apparent Stokes shift of *g*‐*R*‐BINAP is significantly smaller (45 nm / 2720 cm^−1^) compared to crystalline *R*‐BINAP (145 nm / 8250 cm^−1^). This narrowing of the emission band and the decrease in Stokes shift most likely find their origin in the limited excited‐state structural reorganization in the glass. A more rigid local environment and higher barriers associated with disordered packing hinder relaxation of the S_1_ state to its energetic minimum, so that emission occurs from a less stabilized, higher‐energy geometry. This interpretation aligns with FTIR observations (blue‐shift of the phenyl out‐of‐plane deformation mode at 480 to 490 cm^−1^), which point to a stiffer local potential for relevant motions in the glass, and explains the hypsochromic shift in the emission despite the bathochromically shifted excitation of *g*‐*R*‐BINAP.^[^ ^30^, ^39^, ^40^, ^41^ ^]^

Interestingly, literature reports show that BINAP exhibits an even more blue‐shifted emission in solution (e.g., in THF) than in the glass,^[^ ^33^ ^]^ placing the glassy state spectroscopically intermediate between the isotropic solution and the highly ordered crystal. This hierarchy is consistent with the expected influence of environmental C–H···π and π···π interactions and conformational restraints: while in solution the intermolecular π‐interactions between the BINAP molecules are negligible on the timescale of the excited state lifetime, thus mimicking gas phase behavior, the glassy state does allow for some intermolecular interactions and limits conformational freedom. Finally, the crystalline packing allows for optimized C–H···π and π···π interactions with more pronounced conformational freedom in the excited state (see explanations of the spectral band shapes above) and leads to the strongest red shift of the luminescence.

The lifetime of *g*‐*R*‐BINAP has been investigated by time correlated single‐photon counting (TCSPC) and determined to be τ = 1.1 ns, which constitutes an increase of roughly 20% compared to literature‐reported crystalline *R*‐BINAP (τ = 0.8 ns).^[^ ^33^ ^]^ The photoluminescence quantum yield likewise increases from Φ = 0.05 in the crystalline phase to Φ = 0.09 in the glassy state. From these values, the fluorescence radiative rate constant *k*_F_ is estimated to increase by ≈30% from 6.3 × 10^7^ to 8.2 × 10^7^ s^−1^. Again, this enhancement is attributed to the stiffer environment of the glassy state, which suppresses non‐radiative decay channels and ensures more favorable Franck‐Condon factors for the vertical transitions, i.e., increasing oscillator strength for fluorescence in comparison to the crystalline state.

We note that the luminescence lifetime decay of *g*‐*R*‐BINAP shows the presence of a longer‐lived component that could not be precisely specified, but is tentatively attributed to weak phosphorescence. This assignment is supported by spectral deconvolution, where fitting a second Gaussian component beneath the emission band (Figures S19 and S20, Supporting Information) reproduces the phosphorescent contribution previously reported for crystalline BINAP.^[^ ^33^ ^]^

To evaluate the chiroptical emission behavior of the BINAP derivatives, CPL measurements were performed on both crystalline and glassy samples of *R*‐BINAP and *S*‐BINAP (Figure 3c). The corresponding luminescence dissymmetry factors (*g*_lum_, Equation 1) were clearly defined and reliably reflect molecular chirality.

(1) $$g_{\mathrm{lum}}=\frac{2\left(I_{L}-I_{R}\right)}{I_{L}+I_{R}}=\frac{4\;\left|\mu \right|\;\left|m\right|\cos\theta }{{\left|\mu \right|}^{2}+{\left|m\right|}^{2}}$$

where *I*_L_ and *I*_R_ denote the intensities of left‐ and right‐circularly polarized emission, ***µ*** is the electric transition dipole moment, ***m*** the magnetic transition dipole moment, and *θ* the angle between both dipole moment vectors.

In the crystalline state, *R*‐ and *S*‐BINAP exhibit *g*_lum_ values of –1.2 × 10^−3^ and +1.4 × 10^−3^, respectively, demonstrating nearly mirror‐image chiral emission. Strikingly, the glassy forms showed substantially enhanced CPL activity, with *g*_lum_ reaching –0.9 × 10^−2^ for *g*‐*R*‐BINAP and +0.6 × 10^−2^ for *g*‐*S*‐BINAP, values that are considered large for purely organic molecules, where luminescence dissymmetry factors typically range from 10^−4^ to 10^−3^ (**Table** **1**).^[^ ^42^, ^43^, ^44^, ^45^, ^46^, ^47^, ^48^, ^49^, ^50^, ^51^, ^52^ ^]^ The enhancement is plausibly associated with a stiff yet isotropic amorphous environment that reduces coherent intermolecular C–H···π/π···π coupling present in the crystal and diminishes conformational averaging of the emitting state, thereby improving the electronic‐magnetic transition dipole overlap that governs *g*_lum_.^[^ ^53^, ^54^ ^]^ Despite the CPL spectra of the glasses lacking perfect mirror‐image profiles, the opposite signs of *g*_lum_ for each enantiomer confirm the chiral origin of the molecular emission. It is important to note that a large enhancement of the *g*_lum_ values of a chiral molecule typically requires a larger ***m*** and, more importantly, also a reduced ***µ***, because the former is usually 2‐3 orders of magnitude smaller and has a lesser effect on the dissymmetry (see also Equation 1).^[^ ^55^ ^]^ As a consequence, the radiative decay rate *k*_r_ decreases with higher *g*_lum_ values as the Franck‐Condon factors are directly related to ***µ***. However, the glassy state of *R*‐ and *S*‐BINAP leads to a highly unusual situation, where both *k*_r_ and *g*_lum_ are both greatly enhanced.

**Table 1:** Overview of solid‐state CPL‐active purely organic compounds.

Organic compounds	*g*_lum_	Refs.
*R*/*S*‐methyl‐2‐(*9H*‐carbazol‐9‐yl) propanoate	±0.31 × 10^−2^	[50]
pyrene–cyclodextrins (PCDs)	±10^−4^ to ±10^−3^	[49]
*R*/*S*‐4‐bromo‐2‐(((1‐phenylethyl)imino)methyl)phenol	±0.15 × 10^−2^	[44]
*R*/*S*‐tetrasila[2,2]cyclophane derivative 51	±0.17 × 10^−2^	[45]
*R*/*S*‐5.5‐bis(diphenylphosphino)‐4,4′‐bibenzo[d][13]dioxole	±0.12 × 10^−3^	[46]
*S*‐bisimidazolyl (BINOL) dimethyl ether	+0.26 × 10^−2^	[47]
*R*/*S*‐α‐pinene	±0.70 × 10^−2^	[48]
*R*/*S*‐4‐methyl‐2‐(((1‐phenylethyl)imino)methyl)phenol	±0.30 × 10^−2^	[43]
*R*/*S*‐[(2‐dimesitylboryl)phenyl]ethynyl‐substituted [2.2]paracyclophane	±0.54 × 10^−2^	[42]
*R*/*S*‐6,6′‐di(9H‐carbazol‐9‐yl)‐[1,1′‐biphenyl]‐2,2′‐dicarbonitrile	±0.48 × 10^−2^	[51]
*R*/*S*‐octahydro‐binaphthol carbazole	±0.21 × 10^−2^	[52]
*R*‐BINAP	−0.12 × 10^−2^	This work
*g*‐*R*‐BINAP	−0.90 × 10^−2^	This work
*S*‐BINAP	+0.14 × 10^−2^	This work
*g*‐*S*‐BINAP	+0.60 × 10^−2^	This work

**Figure 4:**
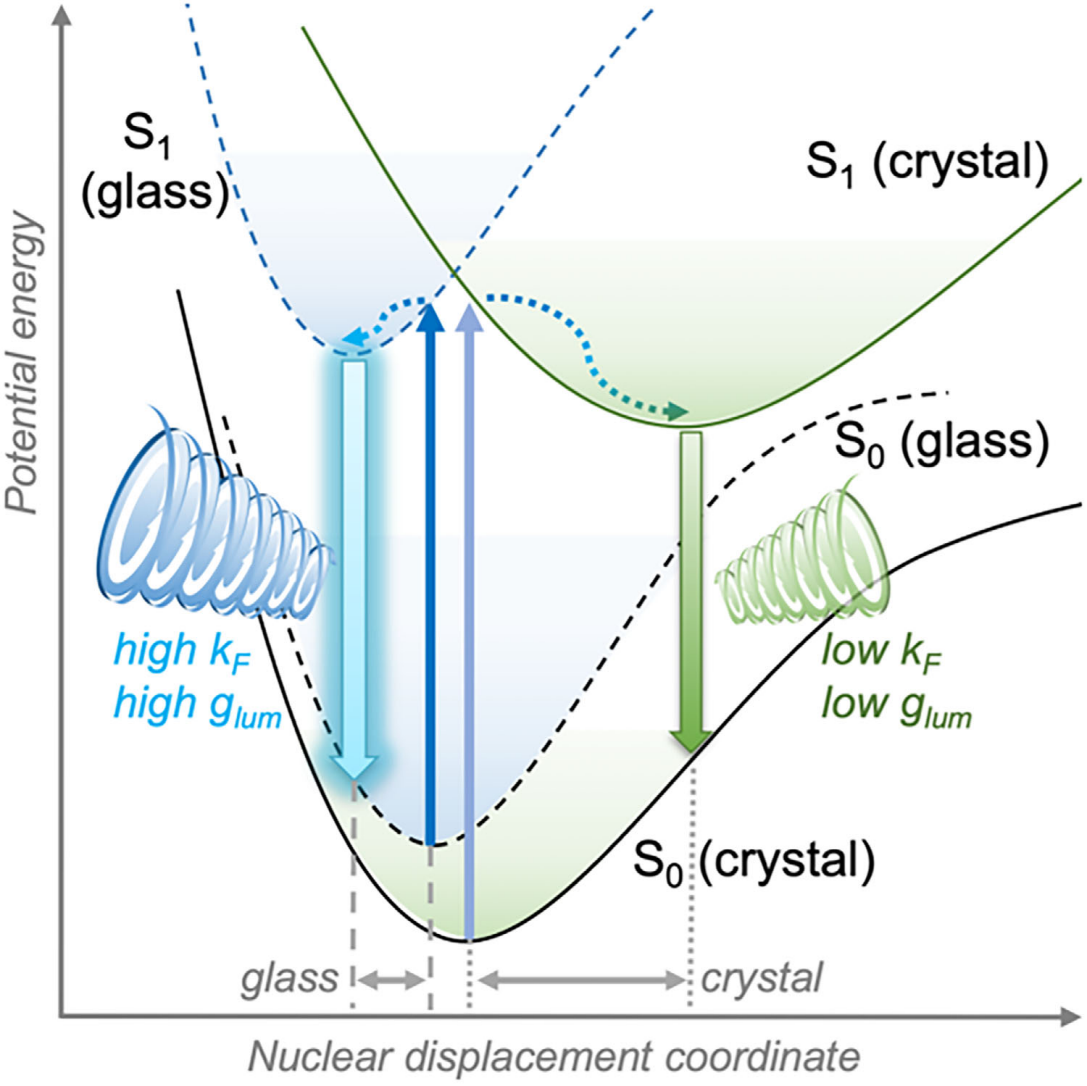
Qualitative energy diagram of crystalline (green solid) and glassy (blue dashed) BINAP, displaying rigidity‐dependent narrowing of the potential energy surfaces of the former, and resulting differences of excitation/emission energies, Stokes shifts, and CPL dissymmetries.

A qualitative energy diagram summarizing the fluorescence pathways in crystal versus glass, consistent with the spectroscopic trends analyzed above (absorption/emission shifts, band narrowing, and elevated *g*_lum_), is shown in **Figure** **4**. Compared to previously reported BINAP‐based CPL systems, often relying on coordination to metal centers or embedding in polymer matrices,^[^ ^37^, ^56^ ^]^ our findings demonstrate that vitrification alone can significantly amplify CPL signals in purely organic materials. This highlights the critical role of solid‐state structural control, even in the absence of crystallinity, in enhancing CPL performance.

The preservation of strong visible emission in the glass, together with its pronounced CPL activity and optical clarity, underscores the promise of chiral BINAP glasses as tunable platforms for photonic and optoelectronic applications. These findings demonstrate that the essential photophysical characteristics of BINAP are retained in the glassy state, with the glass occupying an intermediate spectral position between solution and crystal and revealing the exceptional sensitivity of its optical properties to local environment. These positions melt‐quenched BINAP glasses as a compelling class of materials for future chiral photonic technologies.

## 3 Conclusion

In this study, we have established melt‐quenching as an effective method for preparing optically active glasses from enantiopure BINAP. Systematic investigation of *R*‐BINAP, *S*‐BINAP, and *rac*‐BINAP reveals pronounced differences in glass‐forming ability and photophysical behaviour arising from molecular chirality. Only the enantiopure forms readily yield bulk glasses with glass transition temperatures near 100 °C, while *rac*‐BINAP exhibits a strong tendency toward recrystallization and remains exclusively crystalline under the conditions explored.

Structural analyses by solid‐state NMR, IR spectroscopy, and X‐ray total scattering show that vitrification disrupts long‐range molecular packing but preserves short‐range atomic correlations, with local molecular conformations largely maintained in the amorphous state. CD spectroscopy confirms that the glassy materials remain homochiral, displaying clear mirror‐image signals and only marginal reductions in enantiomeric purity relative to the crystalline materials. Optical measurements demonstrate that the glassy state maintains the characteristic photophysical signatures of enantiopure BINAP, with absorption and emission profiles intermediate between those of the crystal and solution. Notably, the radiative rate constant *k*_F_ is greatly enhanced by ≈30%, while simultaneously CPL activity is also markedly enhanced in the glasses, with *g*_lum_ factors up to an order of magnitude greater than in the crystalline state – among the highest reported for purely organic chiral materials.

These findings position melt‐quenched glasses from chiral organic luminophores as a highly promising platform for advanced optoelectronic materials with tunable chiroptical properties. The combination of homochirality, strong luminescence, and enhanced CPL in an amorphous, processable form opens new opportunities for CPL emission, photonic devices, and chiral sensing. Beyond the specific case of BINAP, this work demonstrates that rational control over glass formation enables the design of functional materials that integrate the versatility of glasses with the specificity of molecular chirality. Looking forward, this strategy can be expanded to other chiral luminophores, with opportunities to further tailor glass properties through functional additives,^[^ ^57^, ^58^ ^]^ to exploit controlled cold‐crystallization for the development of purely organic glass‐ceramics,^[^ ^59^ ^]^ and to optimize processing protocols for next‐generation chiral photonic technologies.

## Conflict of Interest

The authors declare no conflict of interest.

## Author Contributions

N.K. performed PXRD, solution NMR, DSC, TGA, VT‐PXRD, FTIR and CD experiments and data analysis. N.K., C.N. and P.K. performed X‐ray total scattering experiments. N.K. analyzed the XPDF data. C.N. performed DFT calculations. D.D. performed solid‐state NMR experiments with S.V. and R.L. contributing to data analysis and interpretation. P.R. performed the photoluminescence measurements under the supervision of AS. N.K. and S.H. wrote the manuscript with contributions from all the authors. P.R. and A.S. wrote the section on the photoluminescence results. All authors have given approval of the final manuscript.

## Supporting information



Supporting Information

## Data Availability

The data that support the findings of this study are available in the supplementary material of this article.

## References

[1] X. Li , Y. Xie , Z. Li , Adv. Photonics Res. 2021, 2, 2000136.

[2] F. Furlan , J. M. Moreno‐Naranjo , N. Gasparini , S. Feldmann , J. Wade , M. J. Fuchter , Nat. Photon. 2024, 18, 658.

[3] D. G. Suárez‐Forero , M. Jalali Mehrabad , C. Vega , A. González‐Tudela , M. Hafezi , PRX Quantum 2025, 6, 020101.

[4] C. Zhang , X. Wang , L. Qiu , Front. Chem. 2021, 9, 711488. 34568276 10.3389/fchem.2021.711488 PMC8455893

[5] F. Nie , K.‐Z. Wang , D. Yan , Nat. Commun. 2023, 14, 1654. 36964159 10.1038/s41467-023-37331-0 PMC10039082

[6] F. Nie , D. Yan , Nat. Commun. 2024, 15, 9491. 39488522 10.1038/s41467-024-53963-2 PMC11531476

[7] B. Zhou , D. Yan , Matter. 2024, 7, 1950.

[8] Q.‐P. Peng , Z.‐L. He , J.‐H. Chen , J.‐H. Wei , J.‐B. Luo , T.‐C. Wang , K.‐L. Chen , D.‐B. Kuang , Matter. 2025, 8, 102277.

[9] H. Doi , M. Kinoshita , K. Okumoto , Y. Shirota , Chem. Mater. 2003, 15, 1080.

[10] J. C. Ostrowski , J. Hudack , A. Raymond , M. R. Robinson , S. Wang , G. C. Bazan , Chem. ‐Eur. J. 2001, 7, 4500. 11695685 10.1002/1521-3765(20011015)7:20<4500::aid-chem4500>3.0.co;2-v

[11] J. S. Carlson , P. Marleau , R. A. Zarkesh , P. L. Feng , J. Am. Chem. Soc. 2017, 139, 9621. 28632383 10.1021/jacs.7b03989

[12] Y. Xue , Z. Xie , Z. Yin , Y. Xu , B. Liu , Nat. Commun. 2025, 16, 4526. 40374693 10.1038/s41467-025-59787-y PMC12081614

[13] S. F. Swallen , K. L. Kearns , M. K. Mapes , Y. S. Kim , R. J. McMahon , M. D. Ediger , T. Wu , L. Yu , S. Satija , Science 2007, 315, 353. 17158289 10.1126/science.1135795

[14] M. Miyasaka , A. Rajca , M. Pink , S. Rajca , Chem. ‐Eur. J. 2004, 10, 6531. 15540263 10.1002/chem.200400635

[15] G. V. D. Tiers , Thermochim. Acta 1993, 226, 317.

[16] A. Singh , M. K. Jana , D. B. Mitzi , Adv. Mater. 2021, 33, 2005868. 10.1002/adma.202005868 33283383

[17] P. G. Debenedetti , F. H. Stillinger , Nature 2001, 410, 259. 11258381 10.1038/35065704

[18] B. Atawa , N. Couvrat , G. Coquerel , E. Dargent , A. Saiter , Int. J. Pharm. 2018, 540, 11. 29407191 10.1016/j.ijpharm.2018.01.050

[19] A. Singh , D. B. Mitzi , ACS Mater. Lett. 2022, 4, 1840.

[20] C. Ye , L. N. McHugh , P. Florian , R. Yu , C. Castillo‐Blas , C. Chen , A. Lang , Y. Dai , J. Hou , D. A. Keen , S. E. Dutton , T. D. Bennett , Nat. Commun. 2025, 16, 7696. 40825762 10.1038/s41467-025-61410-z PMC12361452

[21] J. E. G. Lipson , Macromolecules 2020, 53, 7219.

[22] T. Koop , J. Bookhold , M. Shiraiwa , U. Pöschl , Phys. Chem. Chem. Phys. 2011, 13, 19238. 21993380 10.1039/c1cp22617g

[23] A. Singh , D. Dayton , D. M. Ladd , G. Reuveni , P. Paluch , L. Montagne , J. Mars , O. Yaffe , M. Toney , G. N. Manjunatha Reddy , D. B. Mitzi , J. Am. Chem. Soc. 2024, 146, 25656. 39230963 10.1021/jacs.4c07411

[24] E. M. Sánchez‐Carnerero , A. R. Agarrabeitia , F. Moreno , B. L. Maroto , G. Muller , M. J. Ortiz , S. de la Moya , Chem. ‐Eur. J. 2015, 21, 13488. 26136234 10.1002/chem.201501178 PMC4567477

[25] J. Kumar , T. Nakashima , T. Kawai , J. Phys. Chem. Lett. 2015, 6, 3445. 26269090 10.1021/acs.jpclett.5b01452

[26] G. Albano , G. Pescitelli , L. Di Bari , Chem. Rev. 2020, 120, 10145. 32892619 10.1021/acs.chemrev.0c00195

[27] A. Miyashita , A. Yasuda , H. Takaya , K. Toriumi , T. Ito , T. Souchi , R. Noyori , J. Am. Chem. Soc. 1980, 102, 7932.

[28] H. Takaya , T. Ohta , N. Sayo , H. Kumobayashi , S. Akutagawa , S. Inoue , I. Kasahara , R. Noyori , J. Am. Chem. Soc. 1987, 109, 1596.

[29] G. Bringmann , A. J. Price Mortimer , P. A. Keller , M. J. Gresser , J. Garner , M. Breuning , Angew. Chem. Int. Ed. 2005, 44, 5384. 10.1002/anie.200462661 16116589

[30] A. M. T. Muthig , O. Mrózek , T. Ferschke , M. Rödel , B. Ewald , J. Kuhnt , C. Lenczyk , J. Pflaum , A. Steffen , J. Am. Chem. Soc. 2023, 145, 4438. 36795037 10.1021/jacs.2c09458

[31] J.‐J. Wang , H.‐T. Zhou , J.‐N. Yang , L.‐Z. Feng , J.‐S. Yao , K.‐H. Song , M.‐M. Zhou , S. Jin , G. Zhang , H.‐B. Yao , J. Am. Chem. Soc. 2021, 143, 10860. 34279083 10.1021/jacs.1c05476

[32] J. Sanz García , C. Lepetit , Y. Canac , R. Chauvin , M. Boggio‐Pasqua , Chem. Asian J. 2014, 9, 462. 25202766 10.1002/asia.201301265

[33] X. Wu , C.‐Y. Huang , D.‐G. Chen , D. Liu , C. Wu , K.‐J. Chou , B. Zhang , Y. Wang , Y. Liu , E. Y. Li , W. Zhu , P.‐T. Chou , Nat. Commun. 2020, 11, 2145. 32358521 10.1038/s41467-020-15976-5 PMC7195388

[34] C. A. Angell , Science 1995, 267, 1924. 17770101 10.1126/science.267.5206.1924

[35] Y. Yue , Front. Mater 2015, 2, 00054.

[36] A. Miyashita , H. Takaya , T. Souchi , Tetrahedron 1984, 40, 1245.

[37] Y. Kono , K. Nakabayashi , S. Kitamura , R. Kuroda , M. Fujiki , Y. Imai , Tetrahedron 2015, 71, 3985.

[38] R. Hamze , S. Shi , S. C. Kapper , D. S. Muthiah Ravinson , L. Estergreen , M.‐C. Jung , A. C. Tadle , R. Haiges , P. I. Djurovich , J. L. Peltier , R. Jazzar , G. Bertrand , S. E. Bradforth , M. E. Thompson , J. Am. Chem. Soc. 2019, 141, 8616. 31062972 10.1021/jacs.9b03657

[39] N. J. Turro , V. Ramamurthy , J. C. Scaiano , Modern molecular photochemistry of organic molecules, University Science Books, Sausalito, Calif, 2010.

[40] M. K. Panda , N. Ravi , P. Asha , A. P. Prakasham , CrystEngComm 2018, 20, 6046.

[41] J. R. Lakowicz , in Principles of Fluorescence Spectroscopy, Springer US, Boston, MA, 2006, pp. 205–235.

[42] L. Guo , M. Zhang , C. Zhao , Molecules 2025, 30, 390. 39860259 10.3390/molecules30020390 PMC11767752

[43] X. Pan , L. Lan , H. Zhang , Chem. Sci. 2024, 15, 17444. 39371458 10.1039/d4sc05005c PMC11447684

[44] X. Pan , A. Zheng , X. Yu , Q. Di , L. Li , P. Duan , K. Ye , P. Naumov , H. Zhang , Angew. Chem., Int. Ed. 2022, 61, 202203938. 10.1002/anie.202203938 35441771

[45] Z. Zhou , L. Gai , L.‐W. Xu , Z. Guo , H. Lu , Chem. Sci. 2023, 14, 10385. 37799998 10.1039/d3sc02690f PMC10548527

[46] P. She , Y. Qin , Y. Zhou , X. Zheng , F. Li , S. Liu , Y. Ma , Q. Zhao , W.‐Y. Wong , Angew. Chem., Int. Ed. 2024, 63, 202403660. 10.1002/anie.202403660 38465907

[47] H. Murata , S. Suzuki , K. Terakubo , Y. Imai , S. Ito , Chem. Asian J. 2024, 19, 202400293. 10.1002/asia.202400293 38750665

[48] B. Zhao , H. Yu , K. Pan , Z. A. Tan , J. Deng , ACS Nano 2020, 14, 3208. 32022541 10.1021/acsnano.9b08618

[49] H. Shigemitsu , K. Kawakami , Y. Nagata , R. Kajiwara , S. Yamada , T. Mori , T. Kida , Angew. Chem., Int. Ed. 2022, 61, 202114700. 10.1002/anie.202114700 34783445

[50] H. Li , H. Li , W. Wang , Y. Tao , S. Wang , Q. Yang , Y. Jiang , C. Zheng , W. Huang , R. Chen , Angew. Chem., Int. Ed. 2020, 59, 4756. 10.1002/anie.201915164 31901181

[51] M. Li , Y.‐F. Wang , D. Zhang , L. Duan , C.‐F. Chen , Angew. Chem., Int. Ed. 2020, 59, 3500. 10.1002/anie.201914249 31872521

[52] Z.‐G. Wu , H.‐B. Han , Z.‐P. Yan , X.‐F. Luo , Y. Wang , Y.‐X. Zheng , J.‐L. Zuo , Y. Pan , Adv. Mater. 2019, 31, 1900524. 10.1002/adma.201900524 31106503

[53] J. Eyyathiyil , S. Ghosh , A. Cheran , S. Geremia , J. Kumar , N. Hickey , P. Thilagar , Commun. Chem. 2025, 8, 126. 40281107 10.1038/s42004-025-01529-8 PMC12032299

[54] Y.‐Q. Zhu , X.‐H. Wang , M.‐X. Wu , Adv. Funct. Mater. 2023, 33, 2308096.

[55] A. M. T. Muthig , J. Wieland , C. Lenczyk , S. Koop , J. Tessarolo , G. H. Clever , B. Hupp , A. Steffen , Chem. ‐Eur. J. 2023, 29, 202300946. 10.1002/chem.202300946 37272620

[56] T. Zhang , Y. Zhang , Z. He , T. Yang , X. Hu , T. Zhu , Y. Zhang , Y. Tang , J. Jiao , Chem. Asian J. 2024, 19, 202400049. 10.1002/asia.202400049 38450996

[57] F. Cao , S. S. Sørensen , A. K. R. Christensen , S. Mollick , X. Ge , D. Sun , A. B. Nielsen , N. C. Nielsen , N. Lock , R. Lu , R. Klemmt , P. K. Kristensen , L. R. Jensen , F. Dallari , J. Baglioni , G. Monaco , M. A. Karlsen , V. Baran , M. M. Smedskjaer , Nat. Commun. 2025, 16, 7001. 40739141 10.1038/s41467-025-62143-9 PMC12311036

[58] P. Kolodzeiski , B. M. Gallant , L. Richter , M. A. T. Ongkiko , C. Franke , A. Kostka , W.‐L. Xue , C. Das , J.‐B. Weiß , E. Kolodzeiski , T. Kress , G. Kieslich , T. Li , A. J. Morris , D. Kubicki , S. Henke , ChemRxiv 2025.

[59] W.‐L. Xue , A. Klein , M. El Skafi , J.‐B. Weiß , F. Egger , H. Ding , S. K. Vasa , C. Liebscher , M. Zobel , R. Linser , J.‐C. Tan , S. Henke , J. Am. Chem. Soc. 2025, 147, 15625. 40270157 10.1021/jacs.5c02767

