# Supplementary material for: Luminescent Chiral Molecular Glasses by Melt‐Quenching Enantiopure BINAP
Source: Adv Sci (Weinh). 2025 Nov 23;13(8):e18879. doi: 10.1002/advs.202518879 (PMC12884765; doi:10.1002/advs.202518879)
Supplement: Supplementary file 1 — Supporting Information [file ADVS-13-e18879-s001.pdf]

Supplementary Information for

Luminescent Chiral Molecular Glasses by Melt-Quenching Enantiopure BINAP

Nuttaporn Krittametaporn[1], Philipp Ralle[1], Dorothea Dierks[2], Christian Nelle[1], Suresh K. Vasa[2], Pascal Kolodzeiski[1], Rasmus Linser[2], Andreas Steffen[1]*, Sebastian Henke[1]*

[1] Anorganische Chemie, Fakultät für Chemie und Chemische Biologie, Technische Universität Dortmund, Otto-Hahn-Straße 6, 44227 Dortmund, Germany, andreas.steffen@tu-dortmund.de, sebastian.henke@tu-dortmund.de

[2] Physikalische Chemie, Fakultät für Chemie und Chemische Biologie, Technische Universität Dortmund, Otto-Hahn-Straße 4a, 44227 Dortmund, Germany

Experimental methods

Preparation of *R*-BINAP and *S*-BINAP glasses

To prevent oxidation, commercial crystalline *R*- and *S*-BINAP were stored in a glovebox immediately upon receipt. All sample preparation was conducted under an inert atmosphere. Crystalline materials were placed into aluminium pans, which were subsequently hermetically sealed. Differential scanning calorimetry (DSC) measurements were performed using a cyclic protocol consisting of two heating scans and one cooling scan. All samples were subjected to the same thermal conditions: heating from room temperature (~25 °C) to 320 °C under N_2 atmosphere at a rate of 10 °C min^{-1}. Following the initial heating, the samples were cooled back to room temperature at a rate of –10 °C min^{-1}, and then reheated to 320 °C. After the thermal treatment, the crucibles were opened under Ar atmosphere in a glovebox. Pale-yellow glasses were obtained and designated *g*-*R*-BINAP and *g*-*S*-BINAP, where "*g*" indicated melt-quenched glass.

Solution ^{1}H and ^{31}P NMR spectroscopy

^{1}H and ^{31}P NMR spectra were recorded on the Bruker Avance Neo 500 MHz spectrometer using deuterated tetrahydrofuran (THF-d_8) as the solvent. NMR spectra were processed using MestReNova software. Chemical shifts in ^{1}H NMR were referenced to the residual proton signals of THF and are reported relative to tetramethylsilane (TMS).

Solid-state ^{31}P MAS NMR spectroscopy

Solid-state NMR experiments were performed on a Bruker Avance NEO spectrometer operating at a ^{1}H Larmor frequency of 700 MHz, using a 2.5 mm TRIGAMMA MAS probe. Samples were packed into 2.5 mm rotors under an oxygen-free atmosphere (glovebox) to prevent oxidative degradation. All spectra were acquired at approximately 20°C with a magic-angle spinning (MAS) frequency of 25.5 kHz. Chemical-shift referencing was achieved externally, using the carbonyl resonance of unlabeled glycine for ^{13}C spectra[1] and NaH_2PO_4 for ^{31}P spectra.[2] Magnetization transfer was achieved through cross-polarization (CP) from ^{1}H to either ^{13}C or ^{31}P nuclei, with proton decoupling during acquisition implemented via the SPINAL-64 sequence[3] at an effective field of 112.6 kHz. All spectra were processed with a 200 Hz exponential linebroadening function using Bruker TopSpin software to enhance the signal-to-noise ratio. Experimental parameters, including number of scans, recycle delays, and acquisition times, are comprehensively documented in Table S2–3. Chemical shifts were

tabulated with multiplicity annotations (s = singlet, m = multiplet) to characterize coupling patterns.

Powder X-ray diffraction (PXRD)

PXRD patterns were recorded using a Bruker D8 Advance diffractometer equipped with Bragg–Brentano geometry and CuKα radiation. Data were collected over a 2θ range of 4° to 50° with a step size of 0.02°. Prior to measurement, samples were finely ground and mounted on a PMMA sample holder with data collected over either 4 or 8 minutes. Structureless profile fitting was carried out using the Pawley method, based on reported crystal structure data[4], as implemented in the TOPAS Academic v6 software.[5]

Variable-temperature (VT-)PXRD

VT-PXRD was carried out in-house at Beamline 9 of DELTA (Dortmund, Germany) using a monochromatic X-ray beam (λ = 0.6199 Å) using a MAR345 image plate detector. Finely ground samples were sealed in borosilicate glass capillaries under an inert atmosphere, then mounted on an Anton Paar DHS1100 hot stage, and heated under a graphite dome. The temperature of the hot stage was calibrated using an integrated thermocouple.

Thermogravimetric analysis (TGA)

TGA measurements were performed on an SDT650 instrument (TA Instruments, USA) under a continuous N_2 flow from 25 °C to 500 °C with a heating rate of +10 °C/min. The sublimation temperature (T_{sub}) was identified as the onset point of significant mass loss in the TGA thermogram. Data analysis was performed using TA Instruments TRIOS software (version 5.1.1.46572).

Differential scanning calorimetry (DSC)

DSC measurements were performed on a DSC25 instrument (TA Instruments, USA). Calibration of temperature and enthalpy was performed using high-purity standards of indium, lead, and tin, while the heat capacity calibration employed a sapphire reference sample. All samples were ground thoroughly and placed in a hermetically sealed aluminium crucible under Ar atmosphere. Measurements were carried out under a constant heating and cooling rate of ±10 °C min^{-1} (except for the calorimetric fragility measurements, see Section S5.3). Data

analysis was performed using TA Instruments TRIOS software (version 5.1.1.46572). The melting temperature (T_m) was identified as the peak temperature of the melting peak, and the glass transition temperature (T_g) was determined from the onset of the corresponding endothermic signal, obtained by applying the standard tangent method.

Fourier-transform infrared (FTIR) spectroscopy

FTIR spectra ($\tilde{v}$ = 400 – 4000 cm^{-1}) were recorded using a Spectrum 3 FT-IR spectrometer (PerkinElmer) equipped with a diamond attenuated total reflectance (ATR) unit operated in reflection mode. Following background correction, powdered samples were placed directly onto the diamond crystal and gently compressed with a stamp to ensure good contact during measurement.

X-ray total scattering

X-ray total scattering data were collected at beamline I15-1 of Diamond Light Source (DLS, UK) using a monochromatic X-ray beam (λ = 0.161669 Å, 76.7 keV). Prior to measurement, finely ground samples were filled into borosilicate capillaries under inert atmosphere. Scattering data from the empty capillary was recorded separately and used for background subtraction. Data processing was performed using the GudrunX software.[6] X-ray pair distribution functions (PDFs), presented as $D(r)$ were obtained by Fourier transformation of the normalized reciprocal space structure data function $S(Q)$.[7]

Circular dichorism (CD) spectroscopy

Circular dichroism (CD) measurements were carried out using a Chirascan qCD spectrometer (Applied Photophysics, UK). Prior to measurement, 2.5 mg of each sample was dissolved in 10 mL of chloroform ($CHCl_3$). A blank spectrum of pure solvent was recorded and used as the baseline. All samples were measured using the same cuvette to minimize instrumental error. Spectra were recorded over the range of 260–500 nm at ambient temperature (~25 °C). CD data were analyzed and processed using the Pro-Data Viewer software.

Photoluminescence (PL) spectroscopy

Excitation and emission spectra were recorded on an Edinburgh Instrument FLS1000 spectrometer, equipped with a 450 W Xenon arc lamp, double monochromators for the

excitation and emission pathways, and a red-sensitive photomultiplier (PMT-980) as a detector. The excitation and emission spectra were corrected using the standard corrections supplied by the manufacturer for the excitation source's spectral power and the detector's sensitivity. Quantum yields of solid samples were measured using an integrating cryosphere (Microstat N2) from Oxford Instruments. The luminescence lifetimes were measured using an EPLED (320 nm), with a TCSPC module. The emission was collected at a right angle to the excitation source. Polarization-sensitive measurements were carried out with EI's CPL option for the FLS1000 (linear polarizer in the excitation beam, photoelastic modulator (PEM) in the emission pathway). The linear polarizer is kept at a 45° angle to the quarter-wave plate in the PEM, which is rotated by 90° with a switching frequency of 50 kHz. The 450 W Xenon arc lamp was used for excitation (for λ_{ex}, see subscript of spectra), intensities of right-hand and left-hand CPL were recorded with an excitation bandwidth of 4.0 nm and emission bandwidth of 4.0 nm, averaged of 150 spectra, and a red-sensitive photomultiplier (PMT-980) as a detector.

Density functional theory (DFT) calculations

Frequency calculations to calculate the IR vibrational frequencies were performed in ORCA (version: 6.0.1)[8] at the B3LYP/def2-TZVP level of theory. An initial geometry of *R*-BINAP was generated using the Python bindings for OpenBabel (openbabel-wheel, version: 3.1.1.21)[9] using a SMILES[10] string (Simplified Molecular Input Line Entry System) and preoptimized using MMFF94[11] (Merck Molecular Force Field) with default settings. The resulting geometry was then optimized in vacuum employing the "TightOpt" keyword, using Grimme's D4 model[12] and the Resolution-of-Identify approximation combined with Chain-of-Spheres Exchange algorithm (RIJCOSX). The auxiliary basis set used was def2/J. The optimized geometry was then used for a frequency calculation at the same level of theory employing the "TightSCF" keyword to confirm convergence by checking for all possible vibrational frequencies. The ORCA .out file of the calculation is given as part of the Supplementary Information.

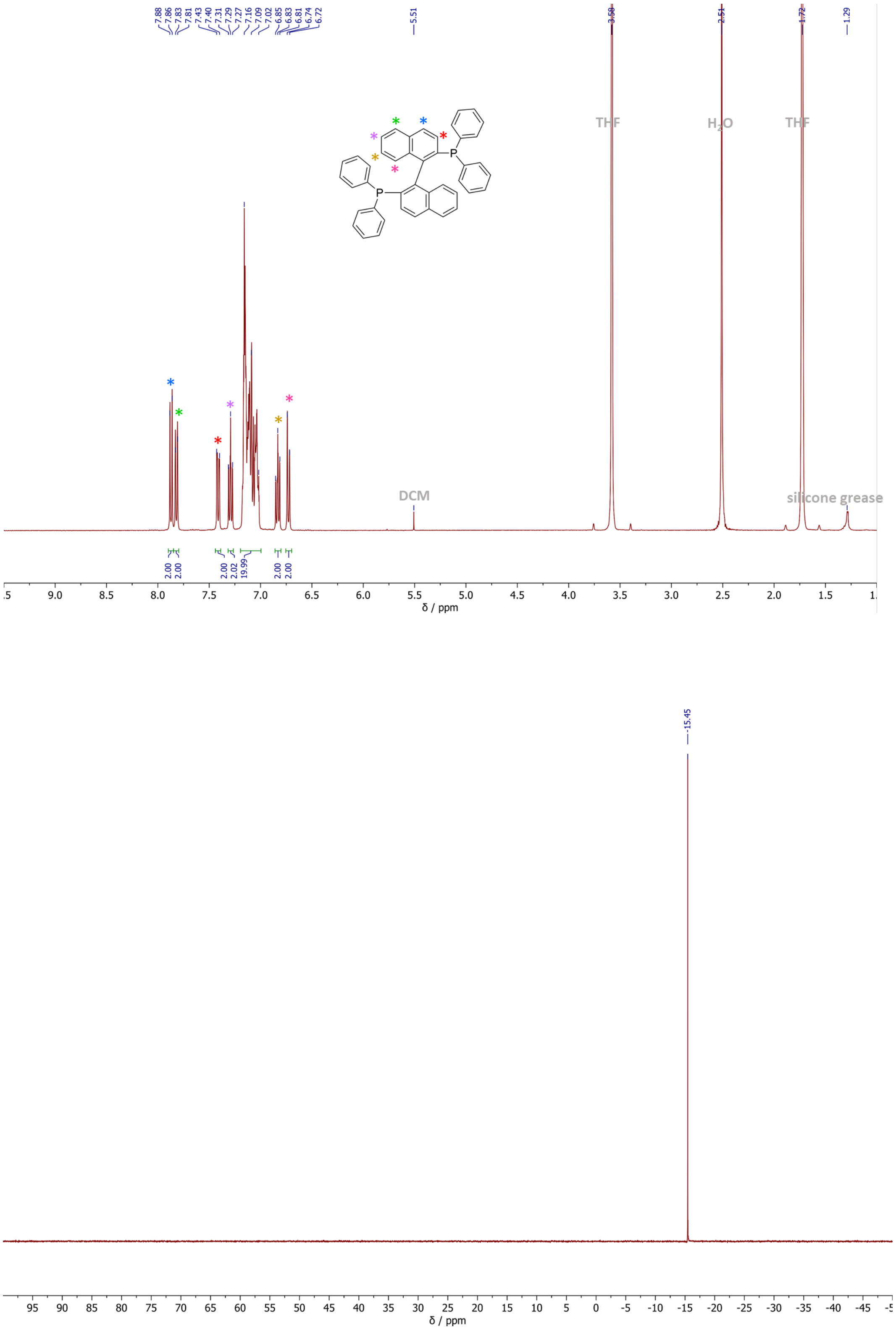

Figure S1. NMR spectra of *rac*-BINAP in THF-d_8: ^{1}H (top) and ^{31}P (bottom).

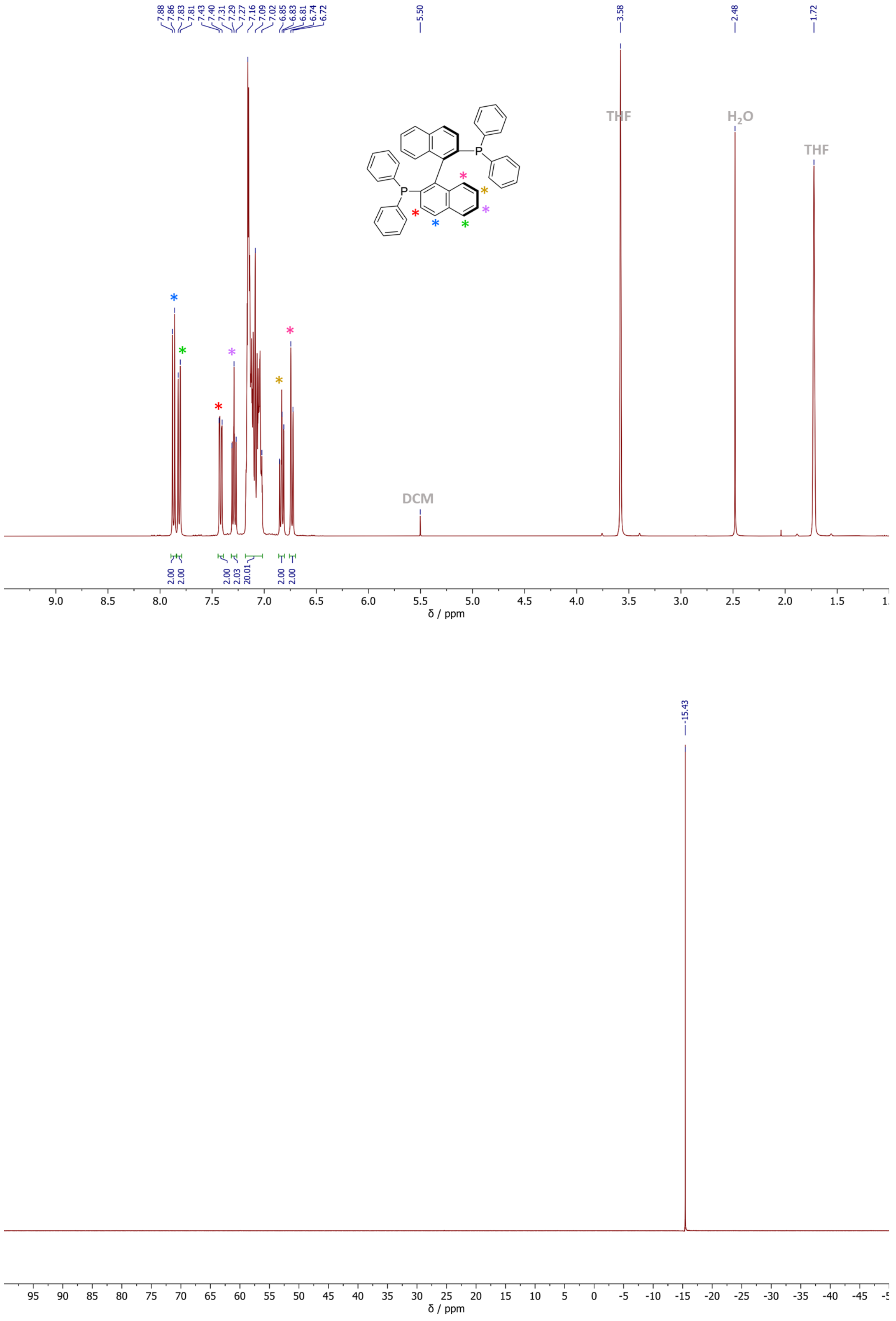

Figure S2. NMR spectra of *R*-BINAP in THF-d_8: ^{1}H (top) and ^{31}P (bottom).

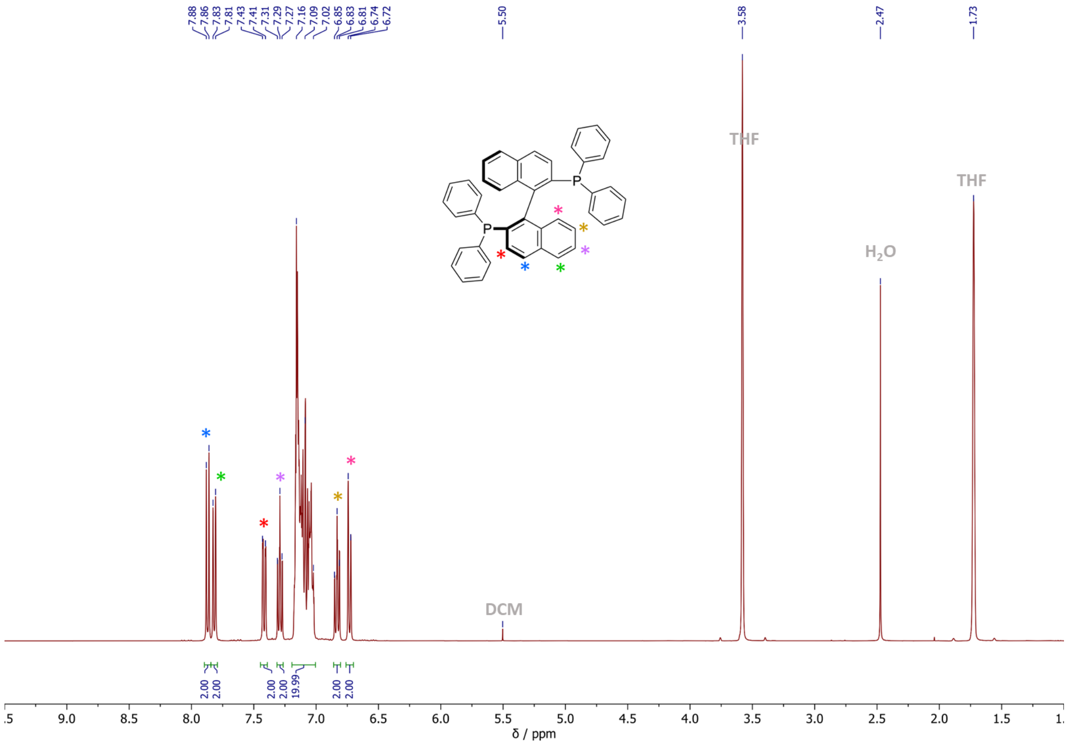

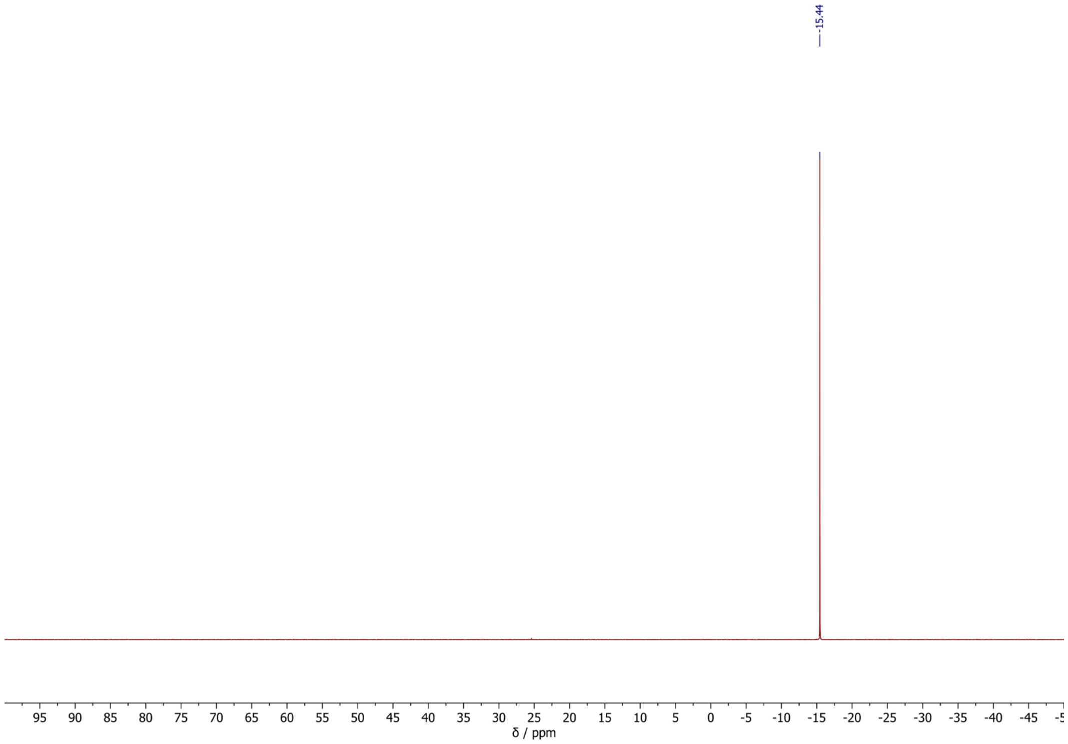

Figure S3. NMR spectra of *S*-BINAP in THF-d_8: ^{1}H (top) and ^{31}P (bottom).

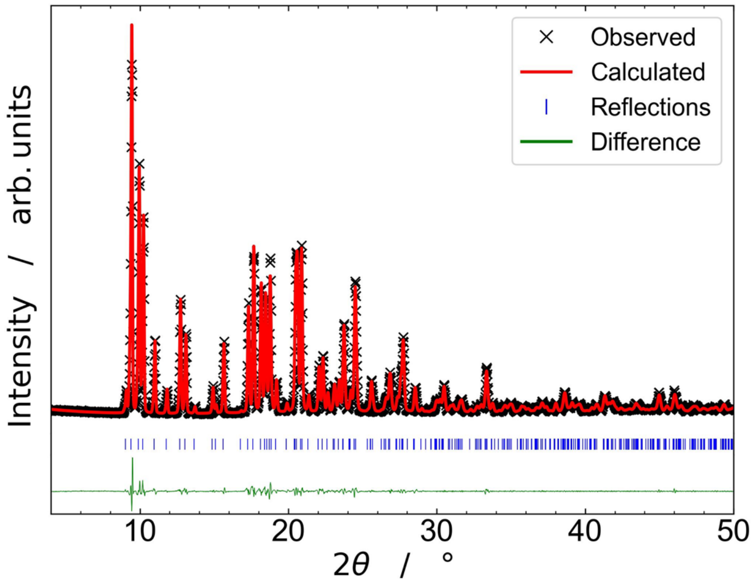

Figure S4. Profile fit (Pawley method) performed on the PXRD patterns of *R*-BINAP using crystallographic parameters from the literature (CCDC: 1968817)[13]. *R*-BINAP crystallize in the monoclinic system, adopting space group $P2_1$.

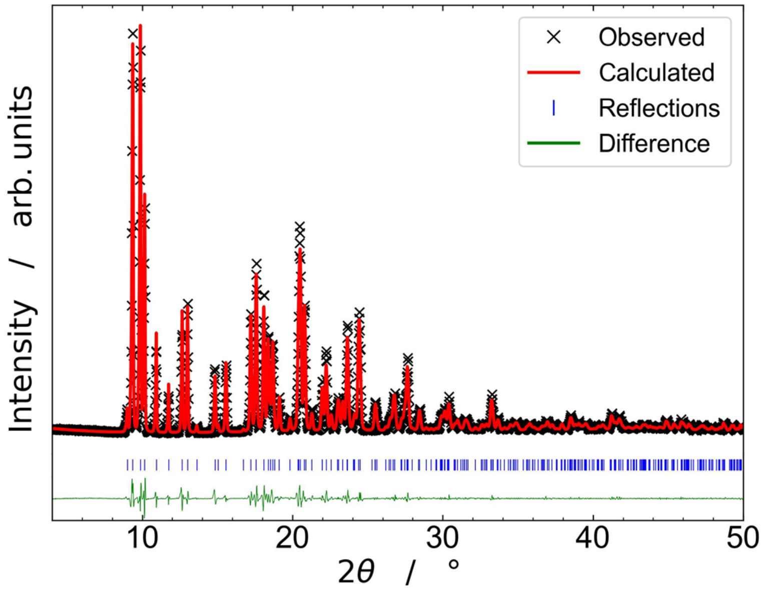

Figure S5. Profile fit (Pawley method) performed on the PXRD patterns of *S*-BINAP using crystallographic parameters from the literature (CCDC: 1968818)[13]. *S*-BINAP crystallize in the monoclinic system, adopting space group $P2_1$.

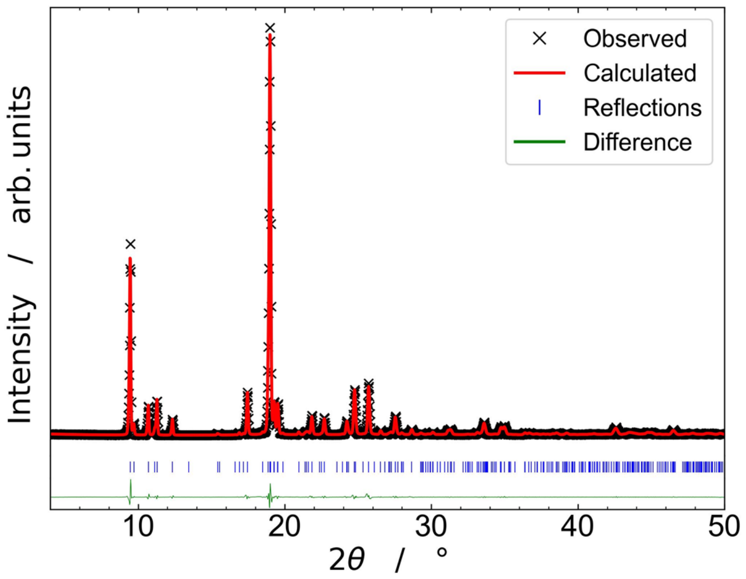

Figure S6. Profile fit (Pawley method) performed on the PXRD patterns of *rac*-BINAP using crystallographic parameters from the literature (CCDC: 1968819)[13]. *rac*-BINAP crystallize in the monoclinic system, adopting space group *C*2/*c*.

Table S1. Crystallographic parameters obtained from profile fitting of the PXRD patterns of the crystalline samples using the Pawley method. The profile fits were performed based on crystallographic parameters reported in the literature.[13]

**Compound**	***R*-BINAP**	***S*-BINAP**	***rac*-BINAP**
crystal system	monoclinic	monoclinic	monoclinic
space group	$P2_1$	$P2_1$	$C2/c$
a / Å	9.1549(10)	9.1891(5)	19.6259(9)
b / Å	18.7816(16)	18.8535(12)	9.2126(8)
c / Å	10.0339(6)	10.0682(5)	19.1225(15)
α / °	90	90	90
β / °	103.269(6)	103.255(5)	107.912(5)
γ / °	90	90	90
V / Å^3	1679.2(3)	1697.8(17)	3289.9(4)
R_{wp}	14.89	11.29	10.41
R_{exp}	3.09	2.95	2.82
χ	4.82	3.82	3.70
Z	2	2	4
V/Z / Å^3	839.6(2)	848.9(9)	822.5(2)
ρ_{cryst} / g cm^{-3}	1.2315(2)	1.2180(12)	1.2572(2)

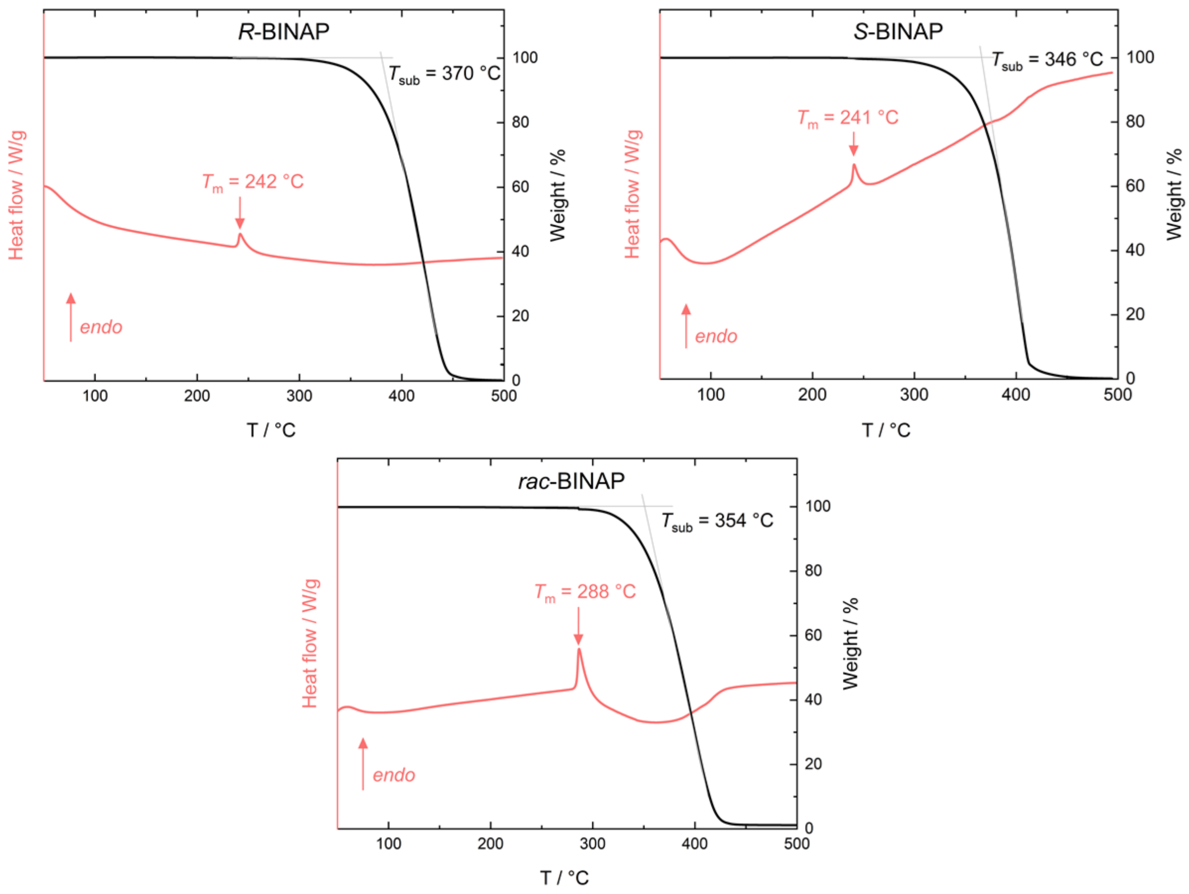

Figure S7. TG/DTA data of commercial BINAP compounds.

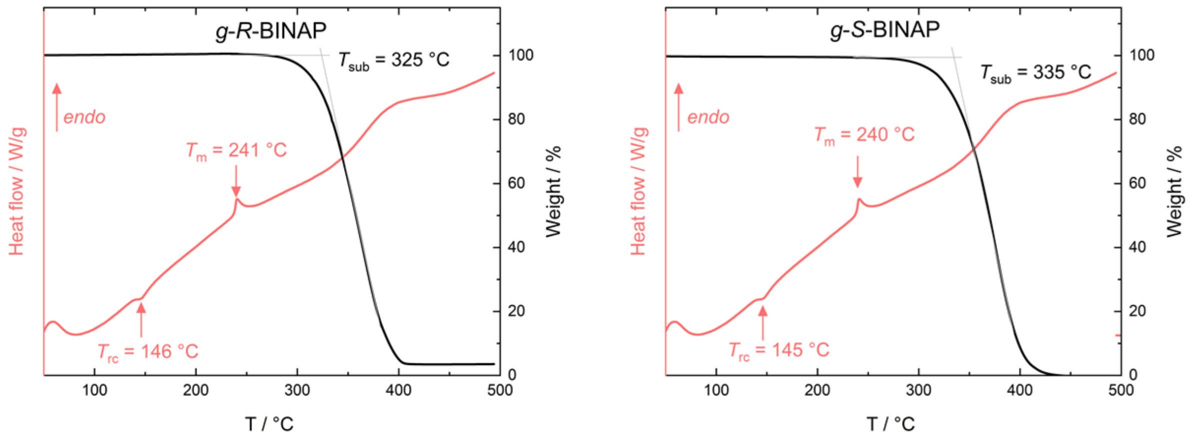

Figure S8. TG/DTA data of *g-R*-BINAP and *g-S*-BINAP. The DTA profiles of both glasses exhibited features indicative of recrystallization and remelting upon heating. The glasses had to be broken into small pieces to be transferred into the alumina crucibles used for the TG/DTA measurements. The presence of partial recrystallisation and melting implies that mechanical forces and decreased particle sizes facilitate recrystallization above T_g.

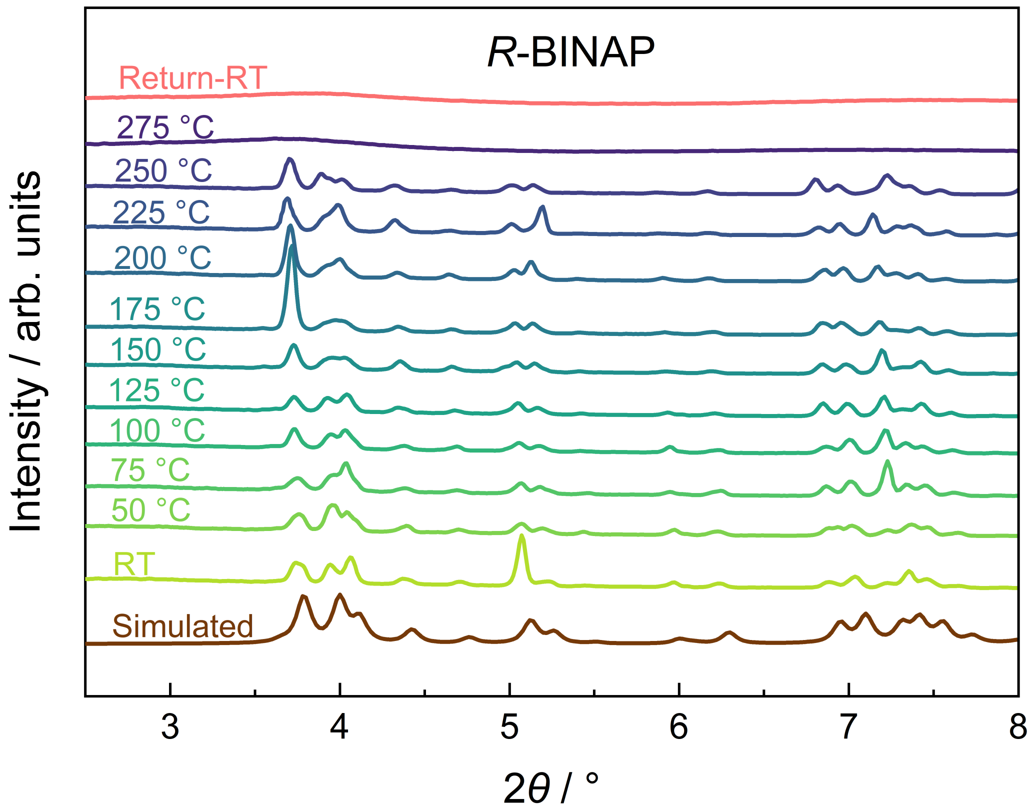

Figure S9. VT-PXRD pattern of *R*-BINAP heating to 275 °C (λ = 0.61992 Å). The calculated PXRD pattern was generated using Mercury software based on the published crystal structure of *R*-BINAP (CCDC: 1968818). Variations in peak intensities with temperature are attributed to the inability to spin the sample capillaries during data collection, resulting in limited powder averaging. Thermal expansion of the powder causes different crystallite orientations to fulfil the reflection condition at each temperature.

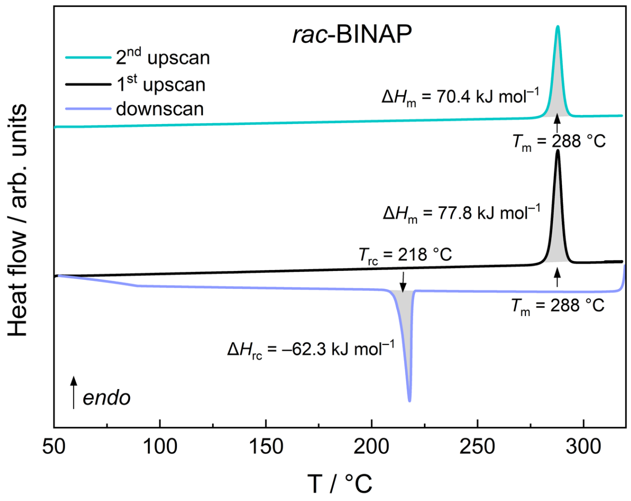

Figure S10. DSC data of *rac*-BINAP (two upscans, one downscan).

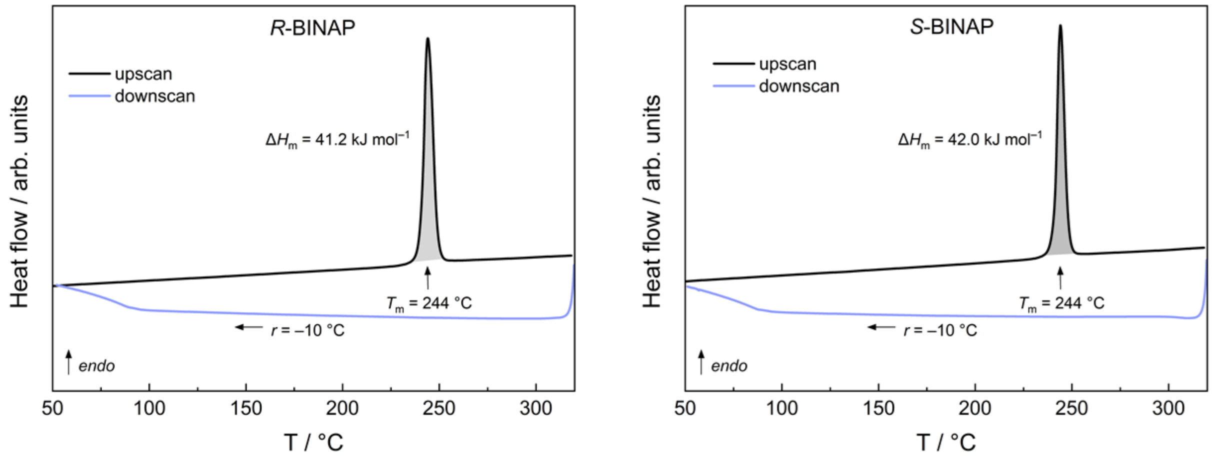

Figure S11. DSC data of enantiopure BINAP derivatives.

Calorimetric fragility assessment

The calorimetric fragilites of the *g*-*R*-BINAP and *g*-*S*-BINAP were determined following procedures reported in a previous study.[14] Melt-quenched *g*-*R*- and *g*-*S*-BINAP samples were subjected to thermal cycling between 50 – 140 °C using various heating and cooling rates ranging from ±25 to ±10 °C min^{-1}. The cooling-rate-dependent fictive temperature (T_f) was obtained with a heating scan subsequent to a previous cooling scan having the cooling rate q_c. The corresponding heating rate of the scan to determine T_f was equal to $-q_c$ of the previous cooling scan. T_f was determined from the onset of the corresponding endothermic signal by applying the standard tangent method. The T_f value at a cooling/heating rate of ±10 °C min^{-1} corresponds to the glass transition temperature (T_g). The calorimetric fragility index *m* was calculated as the slope of the plot of $\log_{10}(1/q_c)$ versus T_g/T_f (with temperatures expressed in Kelvin).

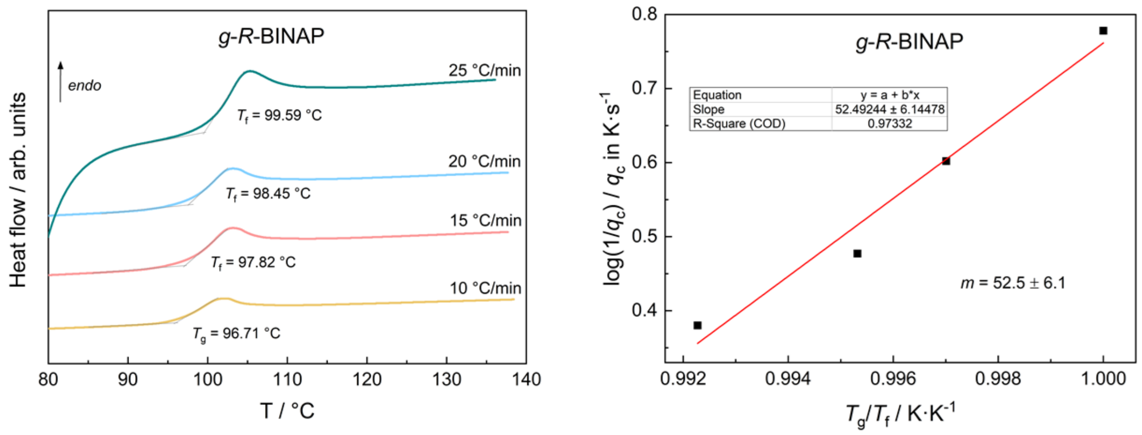

Figure S12. Left: DSC upscans of *g*-*R*-BINAP at heating rates of 10 – 25 °C min^{-1} (previous cooling rates matched). Right: Linear fit for determination of calorimetric fragility index *m*.

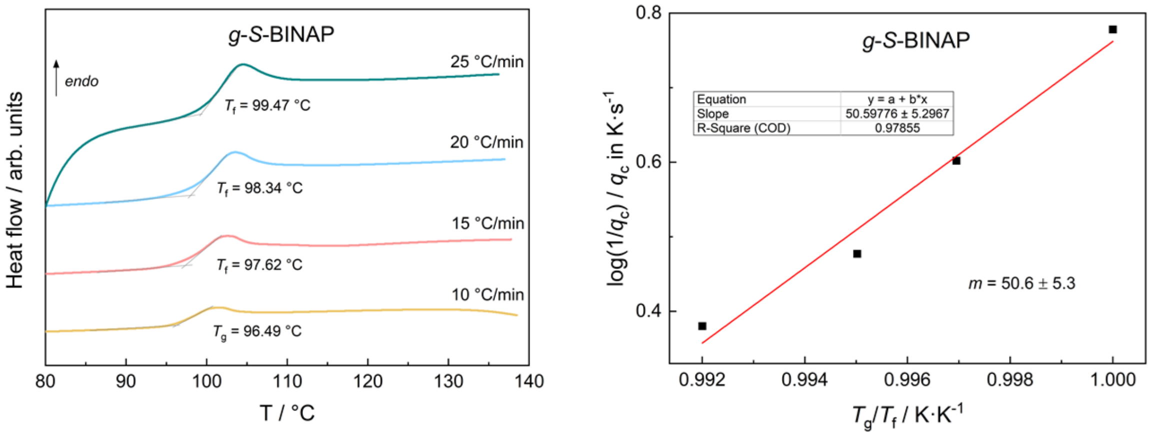

Figure S13. Left: DSC upscans of *g*-*S*-BINAP at heating rates of 10 – 25 °C min^{-1} (previous cooling rates matched). Right: Linear fit for determination of calorimetric fragility index *m*.

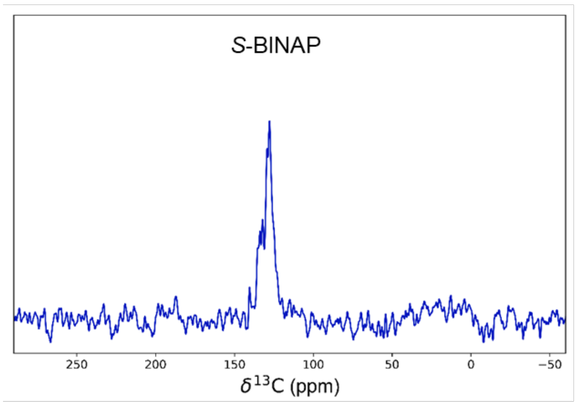

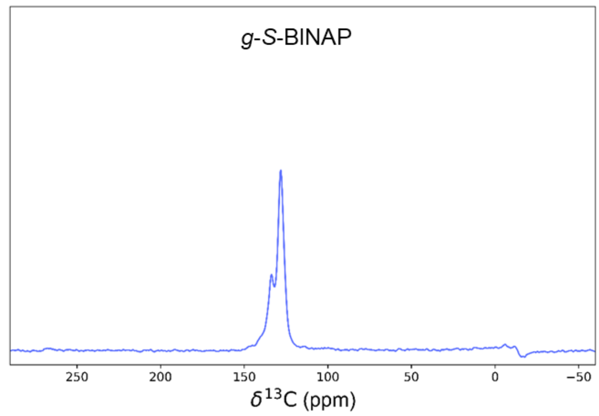

Figure S14. ^{13}C MAS NMR spectra of crystalline *S*-BINAP and its corresponding glass, where the chemical shifts were observed in the range of approximately 142–122 ppm for the crystalline sample and in the range of 153–111 ppm for the glass.

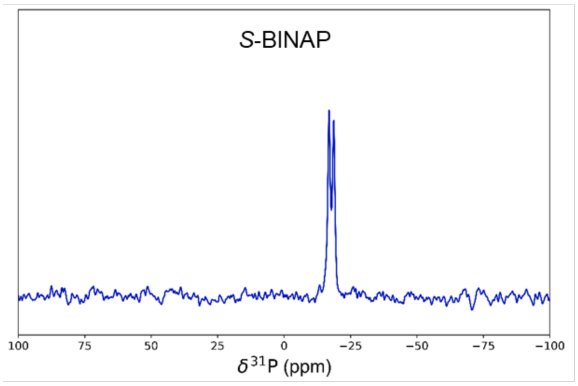

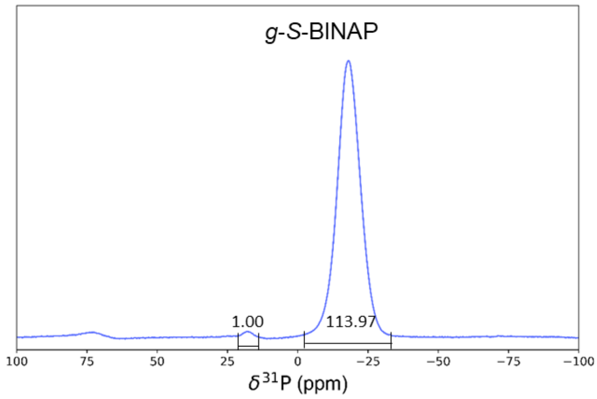

Figure S15. ^{31}P MAS NMR spectra of *S*-BINAP and *g*-*S*-BINAP. For crystalline *S*-BINAP, chemical shifts were observed at –16.91 and –18.62 ppm. In the case of *g*-*S*-BINAP, a main peak appeared at –17.70 ppm, with a broad, weak peak at 18.23 ppm. The calculated ratio of P(V) to P(III) is 0.009.

Table S2. Acquisition parameters and CP conditions used to obtain 1D ^{1}H-^{13}C CP spectra.

Sample	Acquisition parameters			CP conditions				
	Number of scans	Recycle delay /s	Acquisition time F1 /ms	Contact time /μs	RF ^{1}H /kHz	Shape	RF ^{13}C /kHz	Shape
***S*-BINAP**	12288	2.5	19.9	2000	49.5	ramp 80-100	57.1	rectangular
***g*-*S*-BINAP**	2048	2.5	19.9	2000	117.3	ramp 80-100	22.7	rectangular

Table S3. Acquisition parameters and CP conditions used to obtain 1D ^{1}H-^{31}P CP spectra.

Sample	Acquisition parameters			CP conditions				
	Number of scans	Recycle delay /s	Acquisition time F1 /ms	Contact time /μs	RF ^{1}H /kHz	Shape	RF ^{31}P /kHz	Shape
***S*-BINAP**	8192	2.5	14.3	2000	226.6	ramp 80-100	68.1	rectangular
***g*-*S*-BINAP**	2048	2.5	14.3	2000	75.5	ramp 80-100	85.7	rectangular

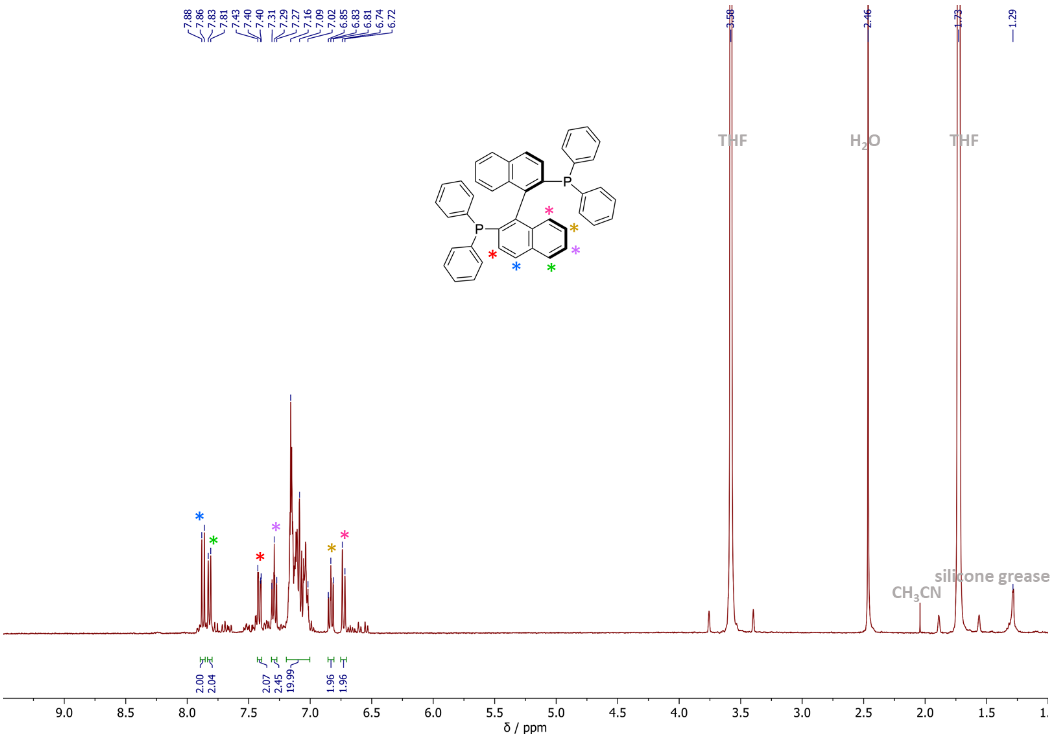

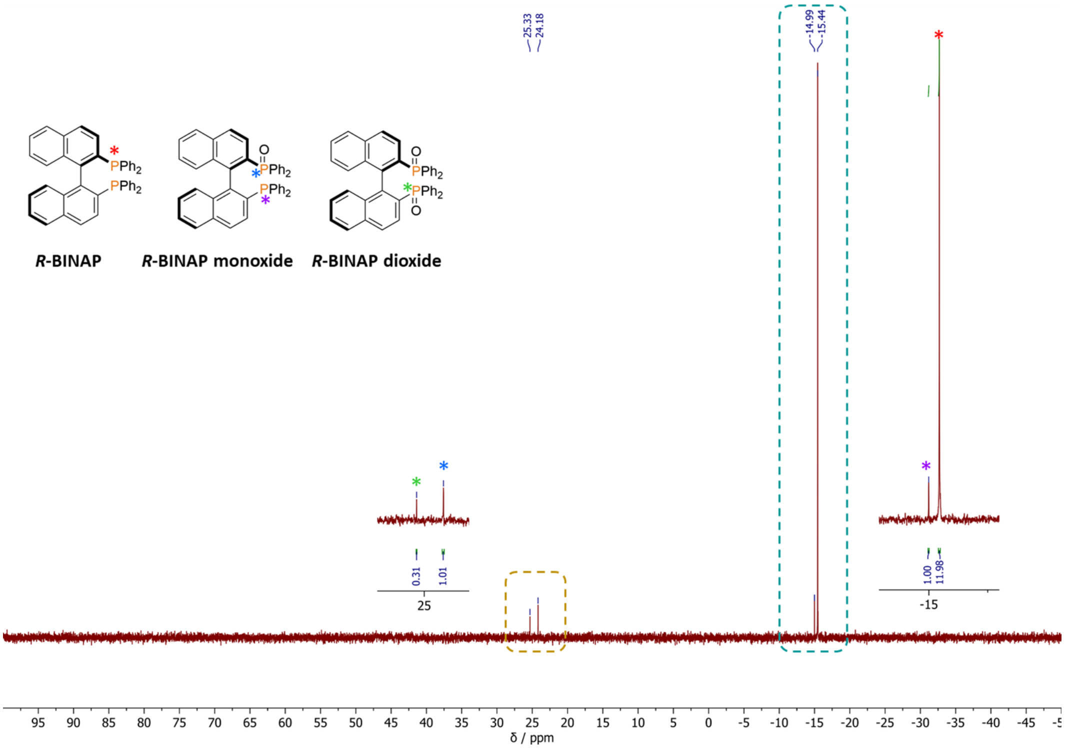

Figure S16. NMR spectra of *g*-*R*-BINAP in THF-*d*8: ^{1}H (top) and ^{31}P (bottom) showing the ratio of P(V) to (PIII) as 0.10.

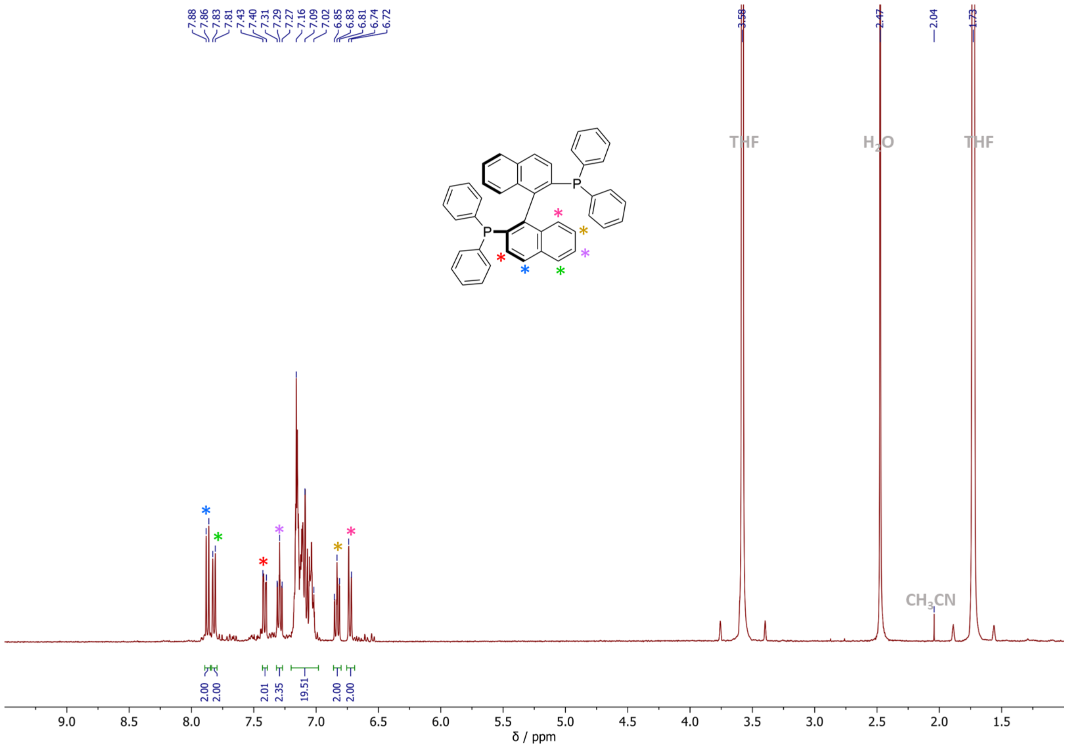

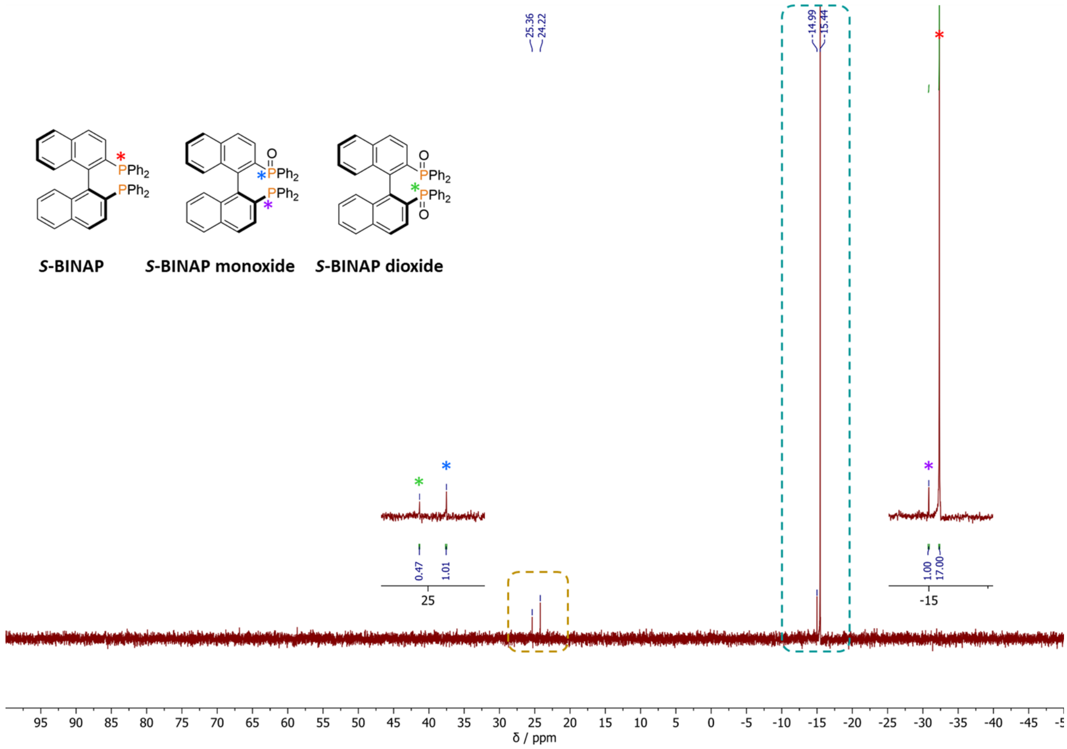

Figure S17. NMR spectra of *g*-*S*-BINAP in THF-d_8: ^1H (top) and ^{31}P (bottom) showing the ratio of P(V) to (PIII) as 0.08.

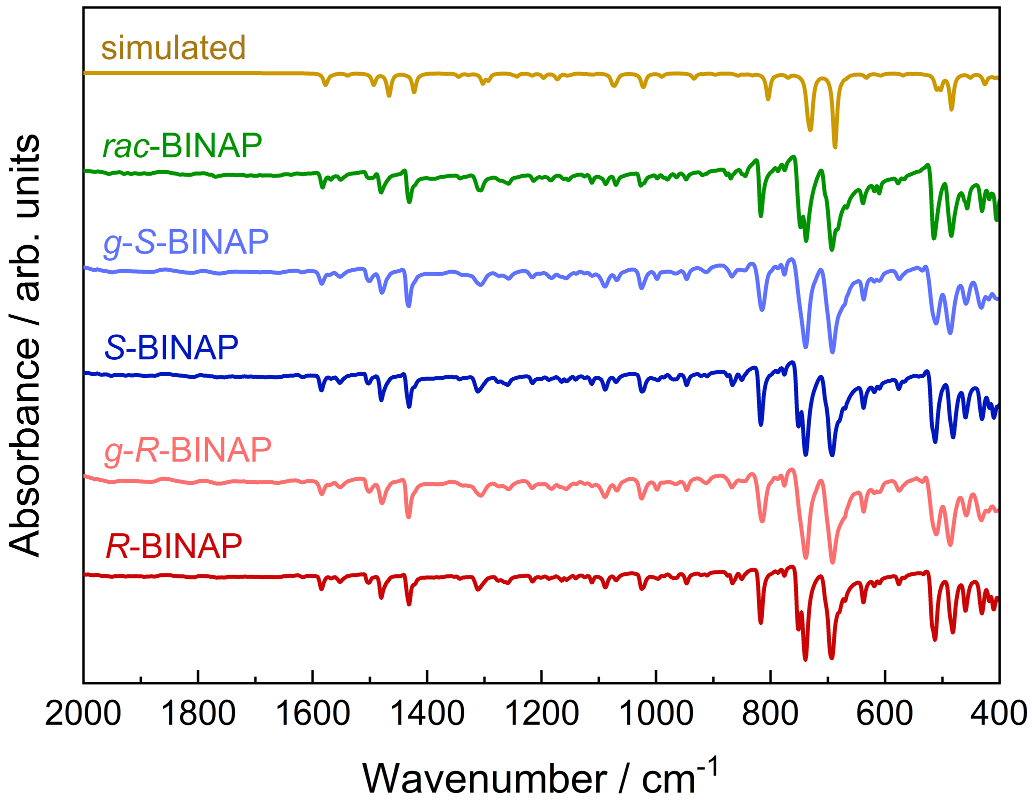

Figure S18. Experimental FTIR spectra of all materials in the range of 400 to 2000 cm^{-1} alongside the simulated spectrum calculated for *R*-BINAP (gas phase) derived from DFT calculations using the ORCA quantum chemistry software.[15]

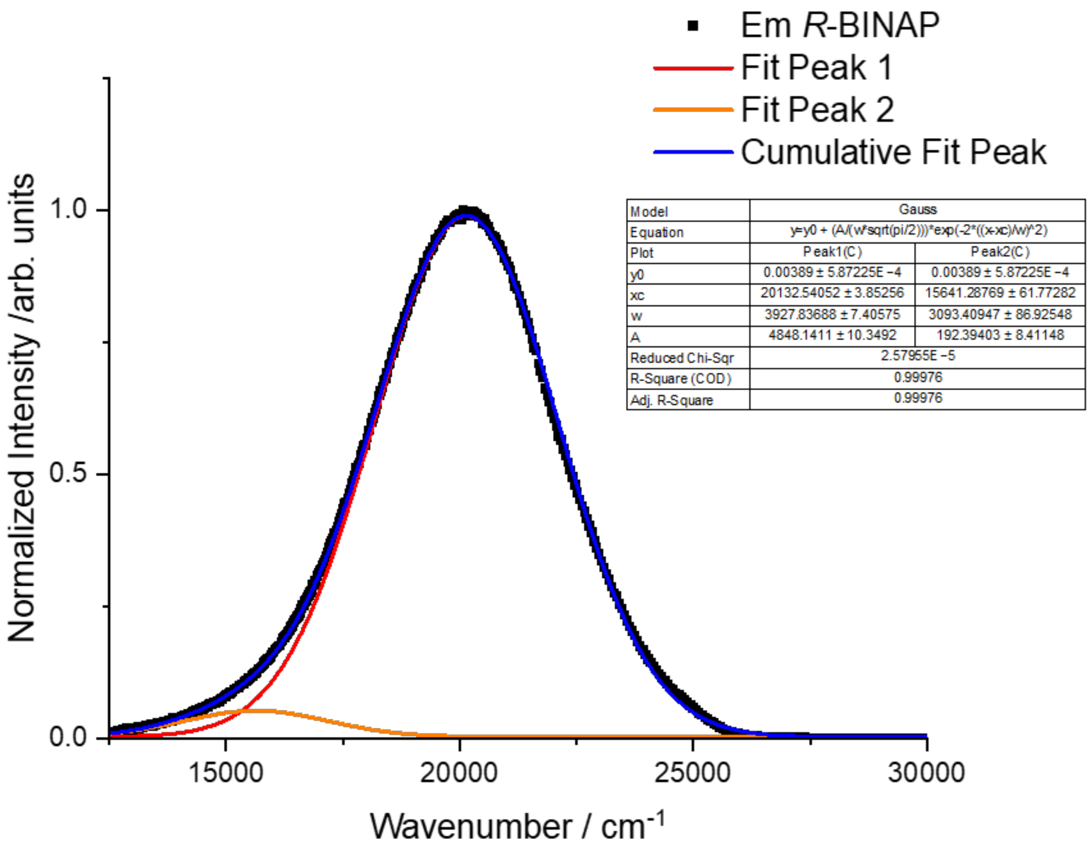

Model	Gauss	
Equation	y=y0 + (A/(w*sqrt(pi/2)))*exp(-2*((x-xc)/w)^2)	
Plot	Peak1(C)	Peak2(C)
y0	0.00389 ± 5.87225E −4	0.00389 ± 5.87225E −4
xc	20132.54052 ± 3.85256	15641.28769 ± 61.77282
w	3927.83688 ± 7.40575	3093.40947 ± 86.92548
A	4848.1411 ± 10.3492	192.39403 ± 8.41148
Reduced Chi-Sqr	2.57955E −5	
R-Square (COD)	0.99976	
Adj. R-Square	0.99976	

Figure S19. Multiple peak Gauss fit of the emission of crystalline *R*-BINAP for fluorescence (red), phosphorescence (orange) and the cumulative emission (blue).

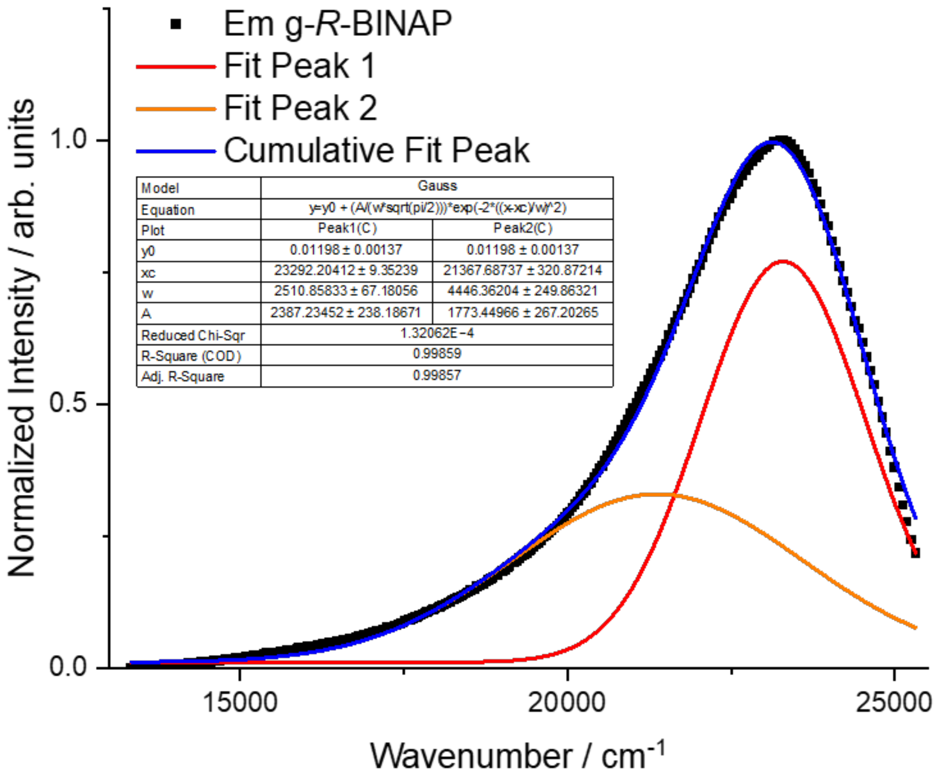

Figure S20. Multiple peak Gauss fit of the emission of *g-R*-BINAP for fluorescence (red), presumed phosphorescence (orange) and the cumulative emission (blue).

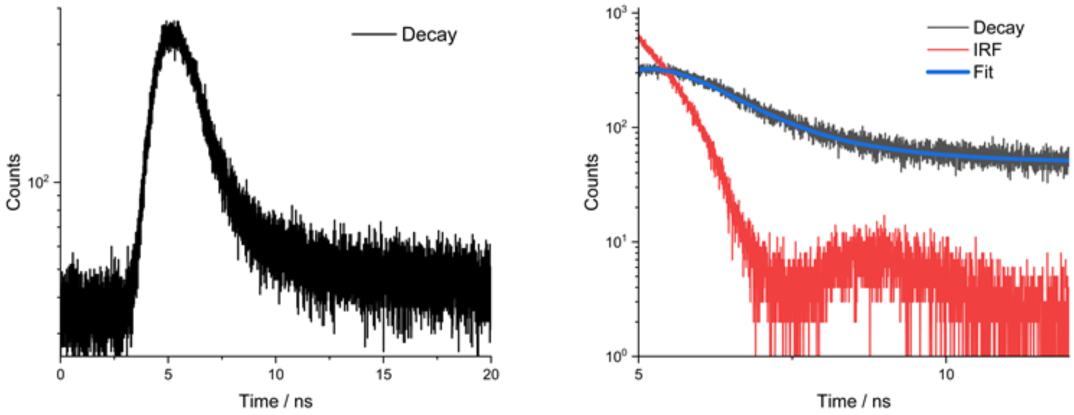

Figure S21. Lifetime decay (left) and reconvolution fit (right) of the photoluminescence lifetime of *g-R*-BINAP after excitation at 320 nm at room temperature.

$$R(t) = e\left(-\frac{t}{\tau}\right)$$

Fitting formula for the lifetime fit, with *t* as the decay time and τ as the lifetime.

Table S4. Parameters of lifetime determination of *g*-*R*-BINAP.

**τ / ps**	**std. dev. / ps**	**χ^2**
1086	11	1.0426

References

[1] C. R. Morcombe, K. W. Zilm, Chemical shift referencing in MAS solid state NMR. *J. Magn. Reson.* **2003**, *162*, 479-486. https://doi.org/https://doi.org/10.1016/S1090-7807(03)00082-X

[2] R. K. Harris, E. D. Becker, S. M. Cabral de Menezes, R. Goodfellow, P. Granger, NMR nomenclature. Nuclear spin properties and conventions for chemical shifts(IUPAC Recommendations 2001). **2001**, *73*, 1795-1818. https://doi.org/doi:10.1351/pac200173111795

[3] B. M. Fung, A. K. Khitrin, K. Ermolaev, An Improved Broadband Decoupling Sequence for Liquid Crystals and Solids. *J. Magn. Reson.* **2000**, *142*, 97-101. https://doi.org/https://doi.org/10.1006/jmre.1999.1896

[4] J. Sanz García, C. Lepetit, Y. Canac, R. Chauvin, M. Boggio-Pasqua, Enantiomerization Pathway and Atropochiral Stability of the BINAP Ligand: A Density Functional Theory Study. *Chem. Asian J.* **2014**, *9*, 462-465. https://doi.org/https://doi.org/10.1002/asia.201301265

[5] A. Coelho, TOPAS and TOPAS-Academic: an optimization program integrating computer algebra and crystallographic objects written in C++. *J. Appl. Crystallogr.* **2018**, *51*, 210-218. https://doi.org/doi:10.1107/S1600576718000183

[6] A. K. Soper, Science, C. Technology Facilities, *GudrunN and GudrunX : programs for correcting raw neutron and x-ray diffraction data to differential scattering cross section*, Science & Technology Facilities Council, Didcot, **2011**.

[7] D. A. Keen, A comparison of various commonly used correlation functions for describing total scattering. *J. Appl. Crystallogr.* **2001**, *34*, 172-177.

[8] F. Neese, F. Wennmohs, U. Becker, C. Riplinger, The ORCA quantum chemistry program package. *J Chem Phys.* **2020**, *152*, 224108. https://doi.org/10.1063/5.0004608

[9] N. M. O'Boyle, M. Banck, C. A. James, C. Morley, T. Vandermeersch, G. R. Hutchison, Open Babel: An open chemical toolbox. *J. Cheminform.* **2011**, *3*, 33. https://doi.org/10.1186/1758-2946-3-33

[10] D. Weininger, SMILES, a chemical language and information system. 1. Introduction to methodology and encoding rules. *J. Chem. Inf. Comput. Sci.* **1988**, *28*, 31-36. https://doi.org/10.1021/ci00057a005

[11] T. A. Halgren, Merck molecular force field. I. Basis, form, scope, parameterization, and performance of MMFF94. *J. Comput. Chem.* **1996**, *17*, 490-519. https://doi.org/10.1002/(SICI)1096-987X(199604)17:5/6<490::AID-JCC1>3.0.CO;2-P

[12] E. Caldeweyher, S. Ehlert, A. Hansen, H. Neugebauer, S. Spicher, C. Bannwarth, S. Grimme, A generally applicable atomic-charge dependent London dispersion correction. *J. Chem. Phys.* **2019***, 150*, https://doi.org/10.1063/1.5090222

[13] X. Wu, C.-Y. Huang, D.-G. Chen, D. Liu, C. Wu, K.-J. Chou, B. Zhang, Y. Wang, Y. Liu, E. Y. Li, W. Zhu, P.-T. Chou, Exploiting racemism enhanced organic room-temperature phosphorescence to demonstrate Wallach's rule in the lighting chiral chromophores. *Nat. Commun.* **2020***, 11*, 2145. https://doi.org/10.1038/s41467-020-15976-5

[14] A. Qiao, T. D. Bennett, H. Tao, A. Krajnc, G. Mali, C. M. Doherty, A. W. Thornton, J. C. Mauro, G. N. Greaves, Y. Yue, A metal-organic framework with ultrahigh glass-forming ability. *Sci Adv.* **2018***, 4*, eaao6827. https://doi.org/10.1126/sciadv.aao6827

[15] F. Neese, The ORCA program system. *WIREs Comput. Mol. Sci.* **2012***, 2*, 73-78. https://doi.org/https://doi.org/10.1002/wcms.81
